# Overexpression of PDSS2-Del2 in HCC promotes tumor metastasis by interacting with macrophages

**DOI:** 10.1038/s41420-024-02274-y

**Published:** 2024-12-18

**Authors:** Guanghui Li, Daqin Suo, Yuanzhen Ma, Tingting Zeng, Jiarong Zhan, Yunfei Yuan, Xin-Yuan Guan, Yan Li

**Affiliations:** 1 State Key Laboratory of Oncology in South China, Sun Yat-Sen University Cancer Center, Guangzhou, 510060 China; 2 Department of Liver Surgery, Sun Yat-Sen University Cancer Center, Guangzhou, China; 3 Department of Clinical Oncology, The University of Hongkong, Hong Kong, China

**Keywords:** Oncogenes, Metastasis

## Abstract

Hepatocellular carcinoma (HCC) is one of the most frequent solid tumors worldwide. According to the Global Cancer Statistics 2020, liver cancer remains the third cause of cancer death globally. Despite significant advances in systemic therapy, HCC still has one of the worst prognoses due to its frequent recurrence and metastasis. Previously we found that PDSS2-Del2 (prenyl diphosphate synthase subunit 2 with exon 2 deletion), a novel variant of PDSS2, could promote HCC metastasis and angiogenesis via activating NF-κB. In this study, we elucidate a novel mechanism by which PDSS2-Del2 enhances HCC metastasis. The overexpression of PDSS2-Del2 in HCC cells promotes the ubiquitination and degradation of SKOR1, consequently heightening SMAD3 phosphorylation. Subsequently, the expression and secretion of MST1 (macrophage stimulatory protein 1) are upregulated, resulting in enhanced recruitment of macrophages into tumor tissues where they differentiate into M2-type macrophages. Co-culture with PDSS2-Del2 overexpressed HCC cells activated the PI3K/AKT signaling pathway in macrophages, and more MMP2 and MMP9 were secreted, which facilitated HCC cell dissemination. Our study elucidates a novel molecular mechanism by which PDSS2-Del2 promotes HCC metastasis, which may contribute to the development of effective HCC clinical treatment and prevent tumor metastasis. Furthermore, MST1 could be a potential therapeutic target, and MST1 inhibitors might be integrated into clinical practice for HCC patients with high expression of PDSS2-Del2.

## Introduction

Hepatocellular carcinoma (HCC) poses a significant global health threat, with its incidence ranking worldwide. Moreover, HCC exhibits a high propensity for metastasis and relapse, yet the underlying mechanisms remain poorly understood, leaving no effective treatment options for metastatic cases [1].

In our previous research, we identified PDSS2 (prenyl diphosphate synthase subunit 2) as a tumor suppressor gene in HCC [2]. Our investigations revealed various splicing variants of PDSS2 within HCC cell lines and tumor tissues [2]. Among these variants, PDSS2-Del2 (prenyl diphosphate synthase subunit 2 with exon 2 deletion) demonstrates completely different functions from PDSS2-full length [2, 3]. It promotes HCC cell metastasis and angiogenesis via activating the NF-κB pathway [3]. Further research demonstrates that PDSS2-Del2 promotes HCC tumor cell metastasis not only by activating the tumor cell itself but also by interacting with immune cells in the tumor microenvironment. In this research, we elucidate a novel molecular mechanism whereby PDSS2-Del2 enhances HCC tumor cell dissemination through its interaction with macrophages within the tumor microenvironment.

## Results

### Higher expression of PDSS2-Del2 is observed in HCC tumor tissues

**Fig. 1:**
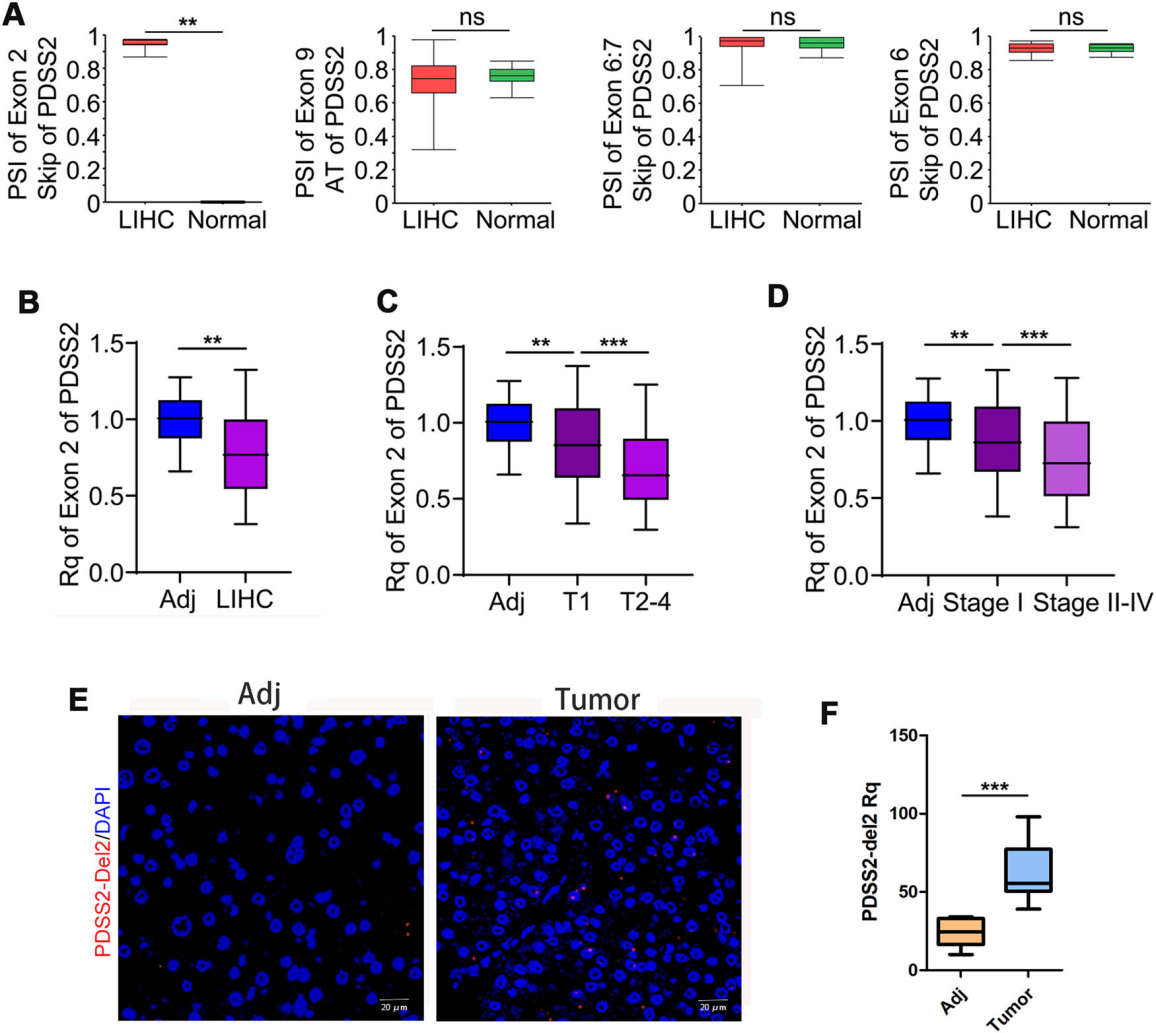
PDSS2-Del2 is upregulated in HCC tumor tissues. **A** The percent spliced index (PSI) of certain alternative splicing of PDSS2 in TCGA-LIHC database; **B**–**D** The relative quantification of PDSS2-exon 2 in LIHC tumor tissues and adjacent tissues (**B**), HCC tumor stages and adjacent tissues (**C**), stage I, stage II–IV, and adjacent tissues (**D**); **E**, **F** FISH results of PDSS2-Del2 in HCC tumor tissues and non-tumor tissues: representative images (**E**) and summary (**F**). (**P < 0.01, ***P < 0.001, unpaired two-sided Student’s *t*-test).

The analysis of TCGA (The Cancer Genome Atlas)-HCC database demonstrated a significantly higher shear rate of the second exon of PDSS2 in tumor tissues compared to normal tissues, suggesting a potential correlation between the deletion of the second exon of PDSS2 and the occurrence of hepatocellular carcinoma (Fig. 1A). To further investigate the relationship between PDSS2-Del2 expression and tumor progression, we evaluated the expression level of the second exon of PDSS2 in adjacent non-tumor tissues, tumor tissues and different stages of tumors. Our findings demonstrated that the expression level of Exon 2 is diminished in HCC tumor tissues compared to adjacent non-tumor tissues (Fig. 1B). Moreover, a reduced expression level of Exon 2 correlates with more advanced malignant clinical stages in patients with HCC (Fig. 1C, D). We also performed fluorescence in situ hybridization on HCC tumor tissues and paired non-tumor tissues. A significantly higher expression level of PDSS2-Del2 was observed in tumor tissues compared with corresponding non-tumor tissues (*n* = 10) (Fig. 1E, F).

### Overexpression of PDSS2-Del2 enhances macrophage migration via MST1

**Fig. 2:**
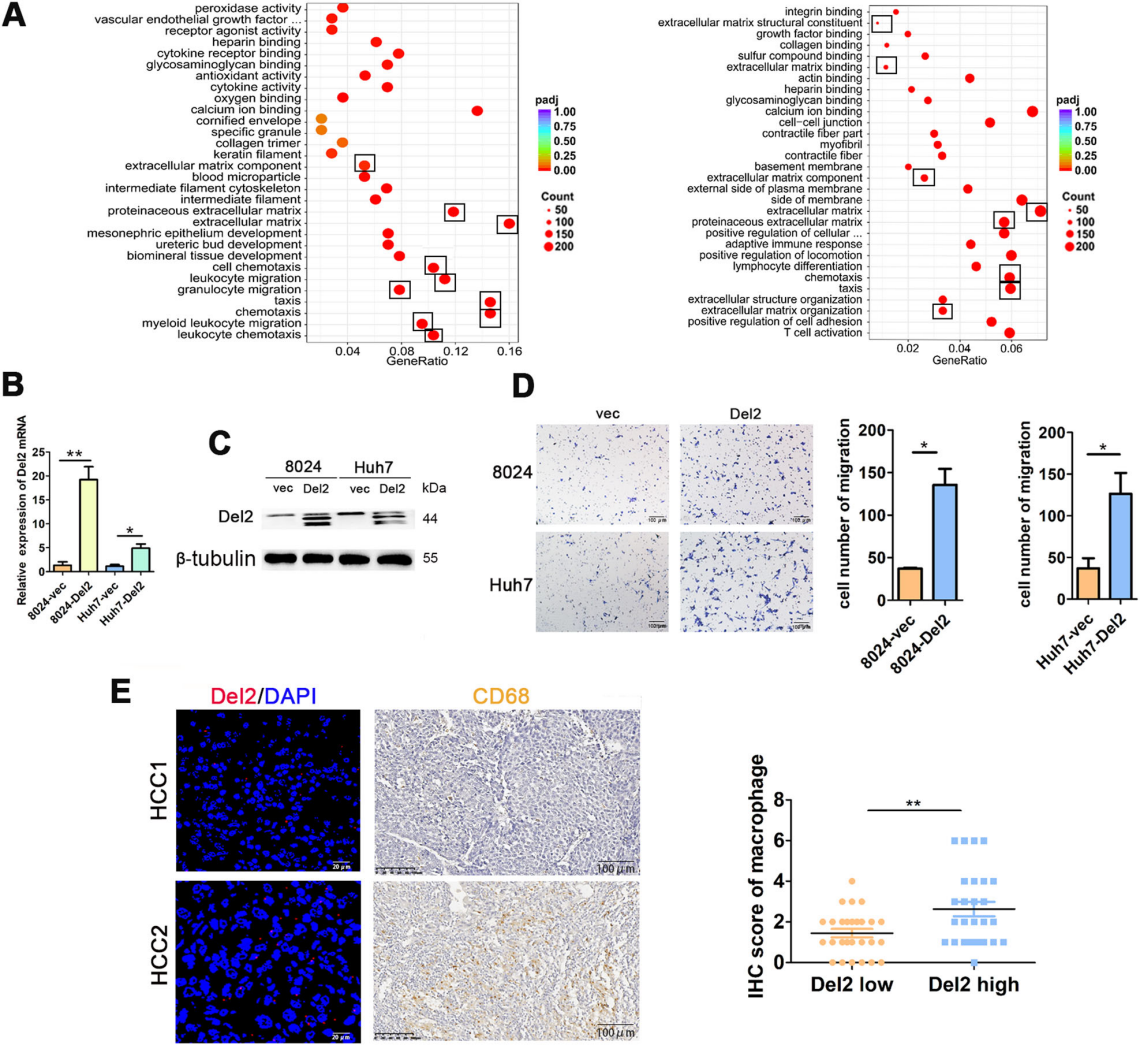
Overexpression of PDSS2-Del2 enhances macrophage migration. **A** GO analysis results of RNA-seq data from tumors formed by PDSS2-Del2 overexpressed HCC cells and control cells in nude mice; **B** The mRNA levels of PDSS2-Del2 in PDSS2-Del2 overexpressed HCC cells and control cells. **C** The protein levels of PDSS2-Del2 in PDSS2-Del2 overexpressed HCC cells and control cells. **D** PMA-treated THP-1 cells were co-cultured with the CM of PDSS2-Del2 overexpressed HCC cells and control cells, and cell migration assay was performed: representative pictures (*left*) and summary (*right*). **E** CD68 staining in PDSS2-Del2 low-expression group and a high-expression group of patients with HCC: representative images (*left*) and summary (*right*). (*P < 0.05, **P < 0.01, ***P < 0.001).

Previous studies have demonstrated that HCC cells with elevated levels of PDSS2-Del2 exhibited increased invasion and metastasis capabilities [3]. Previously we injected HCC cells with PDSS2-Del2 overexpression or vector control cells into nude mice subcutaneously[2]. Tumors from both the experimental and control groups underwent RNA-seq sequencing. GO analysis revealed an enrichment of several pathways associated with tumor metastasis in the experimental group, including extracellular matrix and chemotaxis pathways (Fig. 2A). We focused on chemotaxis pathways for further investigation. Hepatocellular carcinoma cells with PDSS2-Del2 overexpression were co-cultured with macrophages. The results revealed that HCC cells with PDSS2-Del2 overexpression significantly promoted macrophage migration compared to the vector control cells (Fig. 2D). According to the FISH results, the HCC tissue samples were grouped into 2 groups based on PDSS2-Del2 expression levels: a high expression group (>16) and a low expression group (≤16). The infiltration of macrophages in the liver cancer tissues from both groups was assessed using immunohistochemistry. The results showed that the macrophage infiltration in the high-expression group (*n* = 27) was significantly higher than that in the group with low expression of PDSS2-Del2 (*n* = 27) (Fig. 2E).

**Fig. 3:**
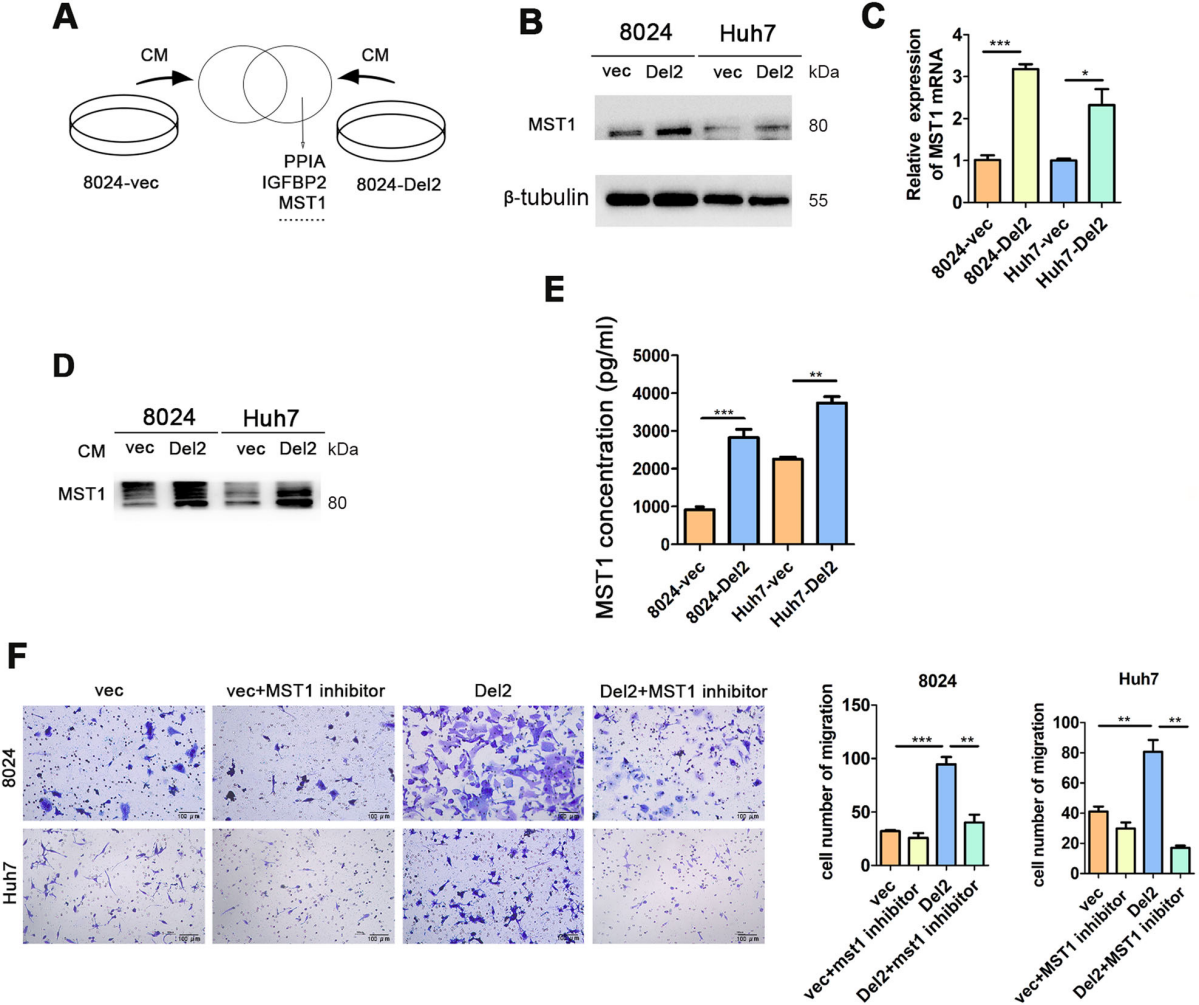
Overexpression of PDSS2-Del2 increases MST1 expression in HCC cells. **A** Schematic diagram of mass spec analysis of CM from 8024-PDSS2-Del2 and control cells. **B**, **C** The protein levels (**B**) and RNA levels (**C**) of MST1 in PDSS2-Del2 overexpressed HCC cells and control cells. **D** The protein levels of MST1 in CM of PDSS2-Del2 overexpressed cells and control cells. **E** MST1 was detected in CM of PDSS2-Del2 overexpressed cells and control cells using ELISA. **F** MST1 inhibitor was added into the co-culture system and migrated HCC cells were assayed: representative images (*left*) and summary (*right*). (*P < 0.05, **P < 0.01, ***P < 0.001).

Next, we collected the conditioned medium (CM) of PDSS2-Del2 overexpressed PLC8024 cells and vector control cells for mass spectrometry analysis (Fig. 3A). Macrophage-stimulating protein (MST1) was detected in PLC8024 overexpressing PDSS2-Del2, but not in the control group. MST1 is a serum protein capable of inducing the spreading, chemotactic migration, and phagocytosis of mouse peritoneal macrophages [4–6]. Western blotting and qRT-PCR were performed on PDSS2-Del2 overexpressed HCC cells and control cells. The protein and RNA levels of MST1 were significantly increased in PDSS2-Del2 overexpressed HCC cells compared with vector control cells (Fig. 3B, C). The conditioned medium was collected, concentrated, and assayed. The MST1 levels in the CM of PDSS2-Del2 overexpressed HCC cells were significantly higher than those in the control cells (Fig. 3D). ELISA results also confirmed that overexpression of PDSS2-Del2 could increase MST1 levels in the CM of HCC cells (Fig. 3E). We treated cells with MST1 inhibitor in the co-culture assay, and the results indicated that MST1 inhibitor significantly impeded macrophage migration in the PDSS2-Del2 group, yet exhibited no impact on the control group (Fig. 3F). These results indicates that overexpression of PDSS2-Del2 promotes macrophage migration via MST1 and the effect could be attenuated by MST1 inhibitor.

### The degradation of SKOR1 leads to increased MST1 expression

**Fig. 4:**
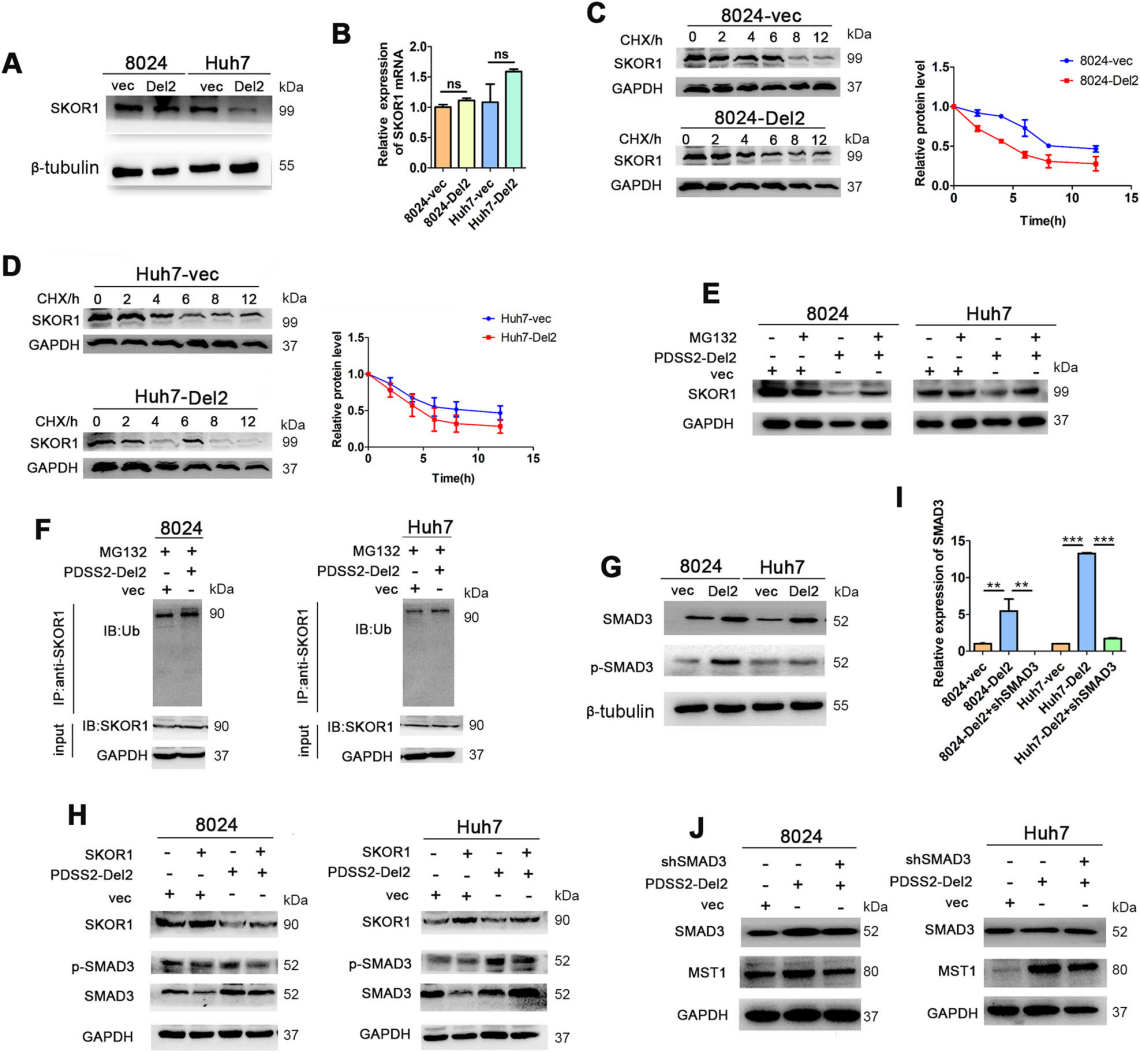
Overexpression of PDSS2-Del2 enhances MST1 expression by promoting SKOR1 degradation. **A** The protein levels of SKOR1 were detected using western blotting. **B** The RNA levels of SKOR1 were detected using qPCR. **C**, **D** The protein levels of SKOR1 were detected in PDSS2-Del2 overexpressed 8024 cells (**C**) and Huh7 (**D**) and control cells. Cells were treated with CHX at various time points. **E** The protein levels of SKOR1 were detected in cells treated with or without MG132. **F** Co-IP was performed with an anti-SKOR1 antibody, and polyubiquitylated SKOR1 was detected by ubiquitin antibody. **G** SMAD3 and p-SMAD3 in PDSS2-Del2 overexpressed HCC cells and control cells. **H** SMAD3 and p-SMAD3 in PDSS2-Del2 overexpressed HCC cells and control cells with or without SKOR1 overexpression. **I** The RNA levels of SMAD3 in SMAD3-KD cells. **J** The protein levels of MST1 in PDSS2-Del2 overexpressed HCC cells with or without SMAD3-KD. (**P < 0.01, ***P < 0.001).

Next, we explored the molecular mechanism by which PDSS2-Del2 overexpression increased MST1 expression in HCC cells. We performed Co-IP experiments with PDSS2 antibodies in 8024 and Huh7 cells, and the pull-down samples were analyzed using mass spectrometry. Among the proteins pulled down by PDSS2 antibody, SKOR1 (SKI family transcriptional corepressor 1) was identified in both 8024 and Huh7 cells with PDSS2-Del2 overexpression. It functions as a transcriptional corepressor of LBX1 and contributes to transcriptional inhibition [7]. We detected the expression level of SKOR1 and found SKOR1 was decreased significantly in PDSS2-Del2 overexpressed HCC cells (Fig. 4A). However, no significant difference was observed in RNA levels of SKOR1 between the experimental and control groups (Fig. 4B). The cells were treated with CHX for varying durations, proteins were harvested and evaluated. The degradation rate of SKOR1 protein exhibited a marked increase in HCC cells overexpressing PDSS2-Del2, relative to the control groups (Fig. 4C, D). It suggests that PDSS2-Del2 overexpression may promote the degradation of SKOR1. In HCC cells overexpressing PDSS2-Del2, SKOR1 protein levels were partially restored following treatment with the proteasome inhibitor MG132 (Fig. 4E). Then proteasome inhibitor MG-132 was used to inhibit the proteolytic process. After 24 h of treatment with MG-132, western blotting results demonstrated a significant increase in SKOR1 ubiquitination in HCC cells overexpressing PDSS2-Del2, compared to control cells (Fig. 4F).

It was reported that SKOR1 could inhibit SMAD3 phosphorylation by binding with SMAD3, thus inhibiting downstream signal transmission [8]. Therefore, SMAD3 was detected and the results exhibited that the protein and phosphorylation levels of SMAD3 were significantly increased in PDSS2-Del2 overexpressed HCC cells compared with control cells (Fig. 4G). The effect was attenuated when SKOR1 was overexpressed (Fig. 4H). These results indicate that SKOR1 can inhibit the phosphorylation of SMAD3. In HCC cells, overexpression of PDSS2-Del2 decreases SKOR1 expression, which subsequently increases phosphorylated SMAD3 levels and influences downstream gene expression. We then knocked down SMAD3 in PDSS2-Del2 overexpressed HCC cells (Fig. 4I), and the results revealed that the expression levels of MST1 were also reduced in SMAD3 knockdown cells (Fig. 4J), which indicates that SMAD3 regulates the expression of MST1. Taken together, these results suggest that the overexpression of PDSS2-Del2 in HCC cells leads to the degradation of SKOR1, an increase in the phosphorylation level of SMAD3, and ultimately, an upregulation in the expression of MST1.

### PDSS2-Del2 promotes the polarization of macrophages towards M2-type

**Fig. 5:**
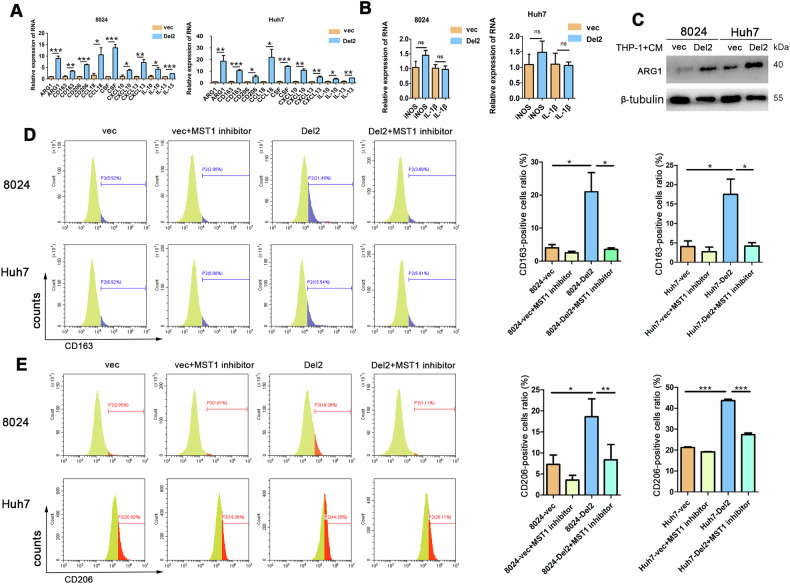
Overexpression of PDSS2-Del2 promotes M2-type polarization of macrophages. **A**–**C** THP-1 cells were co-cultured with CM from PDSS2-Del2 overexpressed HCC cells and control cells. The markers of M2-type (**A**) and M1-type (**B**) were determined using qPCR. The protein levels of ARG1 **C** were determined using western blotting; **D**, **E** THP-1 cells were co-cultured with CM from PDSS2-Del2 overexpressed HCC cells and control cells. The cells were treated with or without an MST1 inhibitor. CD163 (**D**) and CD206 (**E**) were determined using FACS: representative images (*left*) and summary (*right*). (*P < 0.05, **P < 0.01, ***P < 0.001).

Next, we explored the effect of PDSS2-Del2 on macrophage differentiation by co-culturing PDSS2-Del2 overexpressed HCC cells with THP-1 cells. The markers of M1 and M2 macrophages were analyzed using qPCR. The results showed that the M2 markers, including ARG1, CD163, CD206, were elevated in THP-1 cells co-cultured with PDSS2-Del2 overexpressed HCC cells compared to THP-1 cells co-cultured with control cells. However, there was no significant difference observed in M1 markers, including iNOS, IL-1β, between the two groups (Fig. 5A, B). We also tested the protein level of ARG1, which is an important marker of M2 macrophages. The protein level of ARG1 was also increased in THP-1 cells co-cultured with PDSS2-Del2 overexpressed HCC cells (Fig. 5C). To further confirm the results, flow cytometry analysis was also used to examine the differentiation of THP-1 cells co-cultured with HCC cells. As shown in Fig. 5D, E, the ratios of CD163- or CD206-positive cells were significantly increased in THP-1 cells co-cultured with PDSS2-Del2 overexpressed HCC cells. The ratios were reduced when the MST-1 inhibitor was added to the co-culture system (Fig. 5D, E). Overall, these data indicate that co-culturing with PDSS2-Del2 overexpressed HCC cells promotes macrophage differentiation towards the M2 type and that MST1 plays a significant role in this process.

### Co-culturing with PDSS2-Del2 overexpressed HCC cells promotes macrophages to secrete metalloproteinases by activating the PI3K/AKT pathway

**Fig. 6:**
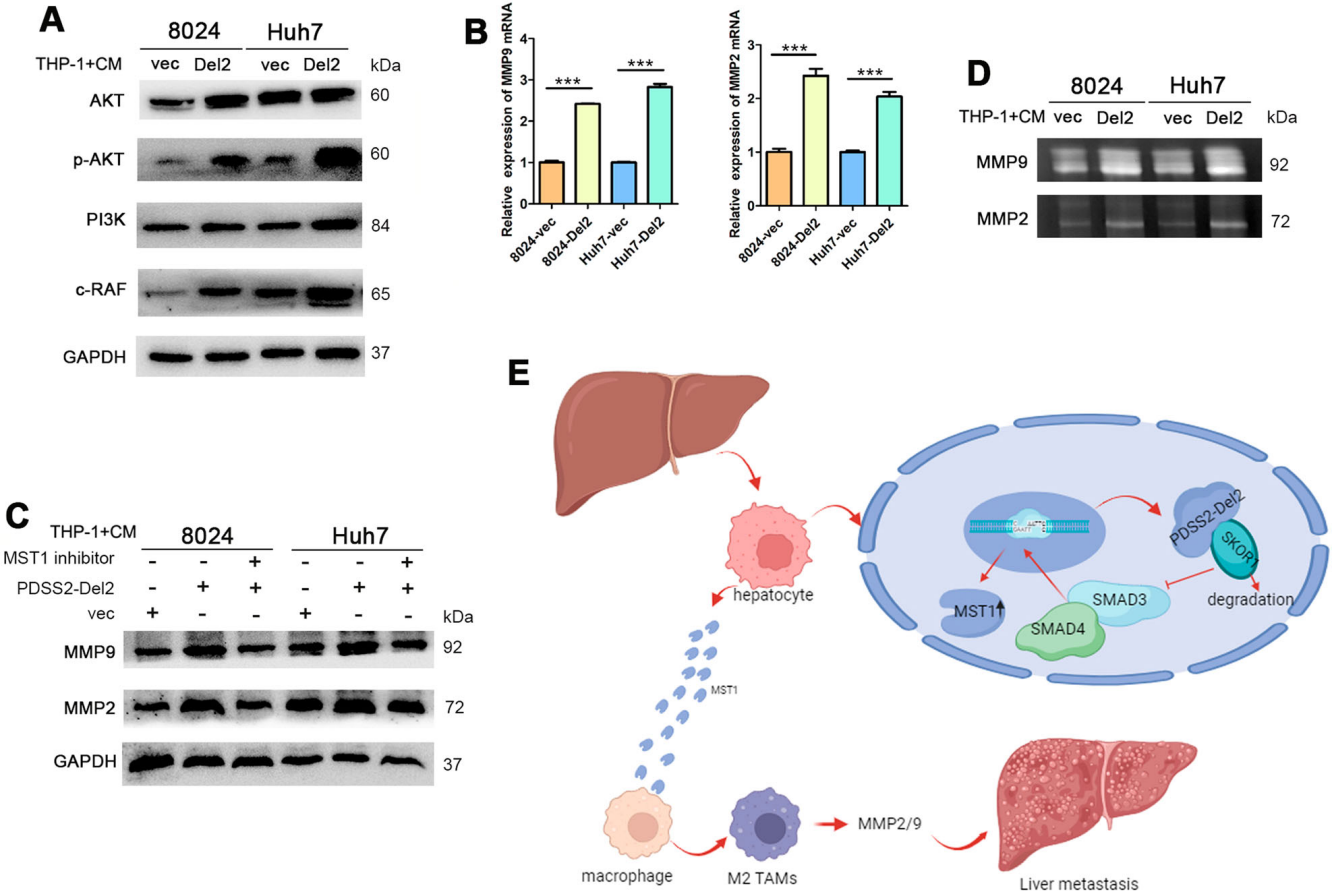
Co-culture with PDSS2-Del2 overexpressed HCC cells promotes macrophages to secrete metalloproteinases by activating the PI3K/AKT pathway. **A** Protein levels of AKT, p-AKT, PI3K, and C-RAF were determined in THP-1 cells co-cultured with CM from PDSS2-Del2 overexpressed HCC cells. **B**, **C** The RNA (**B**) and protein (**C**) levels of MMP9 and MMP2 were determined in THP-1 cells co-cultured with CM form PDSS2-Del2 overexpressed HCC cells and control cells. The MST1 inhibitor (40 nM) was added to the co-culture system (**C**). **D** The activities of metalloproteinases were detected by gel enzyme spectrometry in THP-1 cells co-cultured with CM form PDSS2-Del2 overexpressed HCC cells and control cells. **E** Mechanism by which PDSS2-Del2 overexpressed HCC cells interact with macrophages and enhance tumor cell metastasis.

PI3K (phosphatidylinositol 3-kinase) is a mediator of MST1-RON (macrophage-stimulating protein receptor) [9]. Previous studies have indicated that MST1 can activate the PI3K signaling pathway by inducing tyrosine phosphorylation of the PI3K p85 subunit [10]. The protein levels of PI3K/AKT signaling pathway-related proteins were assayed in THP-1 cells co-cultured with PDSS2-Del2 overexpressed HCC cells and control cells. The protein levels of AKT, phosphorylated AKT, PI3K, and C-RAF were up-regulated significantly in THP-1 cells co-cultured with PDSS2-Del2 overexpressed HCC cells (Fig. 6A). These results indicate that co-culturing with PDSS2-Del2 overexpressed HCC cells activates the PI3K/AKT pathways in macrophages.

It has long been known that elevated activity of various proteolytic enzymes, including zinc-dependent metalloproteinases (MMPs) within the tumor microenvironment, correlate with increased distant metastasis, reduced recovery rates, and decreased overall survival among tumor patients [11]. In co-culturing experiments, macrophages were identified as the main source of MMPs production [12]. The RNA and protein levels of MMP2 and MMP9 were increased in macrophages co-cultured with HCC cells overexpressing PDSS2-Del2, compared to control cells (Fig. 6B, C). When the co-culture system was supplemented with an MST1 inhibitor, both MMP2 and MMP9 levels were decreased (Fig. 6C). Gelatin enzyme assay also demonstrated that the activities of MMP2 and MMP9 were enhanced in macrophages co-cultured with PDSS2-Del2 overexpressed HCC cells (Fig. 6D).

Taken together, these results demonstrate that the overexpression of PDSS2-Del2 in HCC cells enhances MST1 secretion by expediting SKOR1 degradation, subsequently recruiting macrophages into the tumor microenvironment. Co-culturing with PDSS2-Del2 overexpressed HCC cells stimulates the PI3K/AKT signaling pathway in macrophages, promotes macrophage differentiation towards the M2 type, and induces MMP2/MMP9 secretion, thereby providing a suitable microenvironment for tumor metastasis (Fig. 6E).

## Discussion

Hepatocellular carcinoma (HCC) is the fifth most common malignancy and one of the leading causes of cancer-related mortality worldwide [13]. HCC is distinguished by rapid tumor cell proliferation, high metastasis rate, and a significant risk of recurrence. Tumor metastasis persistently presents a significant challenge in the field of cancer therapy, in which the dynamic system of intercellular regulation in the pre-metastatic niche is closely related to tumor progression and distant metastasis [14]. In this study, we demonstrate that high expression of PDSS2-Del2 in HCC is associated with the occurrence and development of hepatocellular carcinoma and promotes tumor metastasis. Importantly, we found that the overexpression of PDSS2-Del2 in HCC cells results in the upregulation of MST1 expression, subsequently recruiting macrophages into tumor tissues, facilitating macrophage differentiation into the M2 type, ultimately establishing a suitable microenvironment for tumor metastasis.

Research has shown that different transcripts of genes play different functions, and the selective splicing of genes is also related to the occurrence and development of various diseases [15]. Our previous studies have found that the new transcript of PDSS2, PDSS2-Del2, is highly expressed in HCC tumor tissues and is correlated with tumor metastasis [3]. While we have observed that PDSS2-Del2 enhances HCC metastasis and angiogenesis by activating the NF-κB pathway, it remains uncertain if additional mechanisms contribute to this process. In this study, we reveal that macrophages in tumor microenvironment also play a significant role in this process. Macrophages can interact with HCC cells with high expression of PDSS2-Del2, thereby promoting the metastasis of HCC cells.

With a better understanding of immune cells and stromal cells within tumor tissues, interest in the tumor microenvironment is increasing. Tumor-associated macrophages (TAMs) are one of the most prevalent cell types in the tumor microenvironment [16]. Recently, the role of TAMs in the process of tumor invasion and metastasis has been a hot issue in oncology research. It has been known for a long time that the co-culturing of macrophages and tumor cells can improve the invasion ability of tumor cells [17]. Many studies have shown that tumor-associated macrophages exhibit the characteristics of M2-like macrophages during tumor progression [18, 19]. They can secrete various cytokines that support tumor cell growth by facilitating angiogenesis, anti-inflammatory responses, immune escape, and other functions [20]. For instance, polarized M2 macrophages can induce a premetastatic niche formation and promote CRLM (colorectal liver metastasis) through CXCL13 secretion [21]. In addition, macrophages can also secrete matrix remodeling molecules to help tumor invasion, such as metalloproteinases and cathepsin. In this study, we demonstrated that the co-culture of PDSS2-Del2 overexpressed HCC cells with macrophages activates the PI3K/AKT pathway in these macrophages. This activation leads to the polarization of macrophages towards the M2 type, increased expression, and secretion of MMP2 and MMP9, thereby reshaping the extracellular matrix and facilitating tumor cell invasion and metastasis. Our study shows that metastasis of hepatocellular carcinoma is a complex process involving multiple molecular interactions of key gene regulation and different cell interactions, which means that multi-target therapy is necessary in clinical practice. It is important to highlight that a combination therapy, such as dimethyl fumarate (DMF) targeting metabolite deficiency [3] combined with an MST1 inhibitor, could potentially enhance the treatment of HCC and inhibit tumor metastasis.

## Conclusions

Overexpression of PDSS2-Del2 in HCC cells leads to SKOR1 ubiquitination and degradation, subsequently increasing phosphorylated SMAD3. This results in enhanced expression of downstream MST1 and its secretion into the extracellular matrix. MST1 then recruits macrophages into tumor tissue and macrophage polarization to M2 type, which secrete MMP2 and MMP9 to promote HCC cell dissemination. Overall, our study elucidates the molecular mechanism through which PDSS2-Del2 promotes HCC metastasis, potentially contributing to the development of effective clinical treatments for HCC and preventing tumor metastasis. Furthermore, MST1 could serve as a potential therapeutic target, and an MST1 inhibitor might be integrated into clinical practice for HCC patients with high PDSS2-Del2 expression.

## Materials and methods

### Cell culture

Hepatoma cell lines PLC8024, Huh7, and human peripheral blood Monocyte cell line THP-1 were obtained from the Institute of Virology, Chinese Academy of Medical Sciences (Beijing, China). Cytogenetics confirmed that PLC8024, Huh7, and THP-1 cells were of human origin. Two hepatocellular carcinoma cell lines were cultured with DMEM containing 10% FBS, and THP-1 was cultured with RPIM1640 containing 10% FBS. All cells were incubated in a humidified incubator containing 5% CO_2_ at 37 °C, and all cells were negative for mycoplasma contamination.

### Patients and tissue samples

HCC tumor tissues and corresponding non-tumor tissues were obtained from the Sun Yat-sen University Cancer Center (SYSUCC). The clinical specimens used in this study were approved by the Sun Yat-sen University Cancer Center Human Research Ethics Review Committee. The patient’s written informed consent was obtained, and the study was conducted in accordance with the Declaration of Helsinki.

### Fluorescence in situ hybridization (FISH) assay

D-T-R mRNA in situ hybridization detection kit was purchased from EXONBIO (Guangzhou, China). The experiment was carried out according to the instructions. The paraffined specimens were dewaxed and rehydrated, followed by pre-hybridization and hybridization with a probe. The slides were counterstained with DAPI and the images were taken under the fluorescence microscope. The sequence of probe specifically targeting PDSS2-Del2 mRNA:

5′-CAAACTTCTTTGACTGGCTGTGGTAAGCAG-3′.

### Plasmid and lentivirus transduction

Plenti6-PDSS2-Del2 and the pLKO.1-scramble-shRNA targeting SMAD3, as well as the corresponding control plasmids, were purchased from GeneCopoeia (Guangzhou, China). For lentiviral production, the 293FT cells (Invitrogen, Carlsbad, CA) were co-transfected with the PMD2G, psPAX2 (gag, pol), and Plenti plasmids. The cell lines were transduced with lentivirus, and stable cells were established by puromycin (Sigma-Aldrich, Shanghai, China) selection.

The sequence of short hairpin RNA (shRNA) specifically targeting SMAD3: 5′-GCGTGAATCCCTACCACTACC-3′.

### RNA isolation and qRT-PCR

Total RNA was isolated from cells using TRIZOL Reagent (Invitrogen, Carlsbad, CA) followed by reverse transcription using the Evo M-MLV RT kit with gDNA for qPCR II (Accurate Biology, Hunan, China). The mRNA levels were detected by qRT-PCR with SYBR Green on Roche LightCycler480 (Roche, Basel, Switzerland). 18 s was used as an endogenous control. The primer sequences used were listed in the Supplementary Materials.

### Western blotting assays

Western blotting analyses were conducted following the established protocol. Briefly, total proteins were extracted from cells using RIPA lysis buffer (CST, Danvers, MA) supplemented with complete protease inhibitors and phosphatase inhibitors (Merck, Darmstadt, Germany). Whole-cell lysates were subsequently separated by electrophoresis with sodium dodecyl sulfate (SDS) polyacrylamide gel and then transferred to PVDF membranes (Millipore, Bedford, MA). The membrane was blocked with 5% bovine serum albumin (BSA) at room temperature for 1 h, followed by incubation with the primary antibody at 4 °C overnight and then the secondary antibody at room temperature for 1 h. The antibodies used were listed in the Supplementary Materials.

### Immunohistochemistry (IHC)

IHC was performed according to a standard streptavidin-biotin-peroxidase complex method. Paraffin-embedded tissue sections were dewaxed with xylene and blocked with 3% hydrogen peroxide solution. The slides were incubated overnight with primary antibody (anti-CD68: 1:1000) (CST, Danvers, MA) at 4 °C. The proportion of immune-positive cells was scored on a scale of 0–4 (<5%-0, 5–25%-1, 26–50%-2, 51–75%-3, and >75%-4). The staining intensity was scored as negative (0), weak (1), medium (2), and strong (3). The IHC score was determined by multiplying the proportion of immune-positive cells with the intensity score. Two investigators, who were unaware of the group allocation, scored the degree of immunostaining.

### Migration assays

Migration assays were performed using transwell chambers with 8 μm PET membrane (Corning, Corning, NY). In short, THP-1 (1 × 10^5^ cells) was seeded in 200 μl RPMI1640 medium in the upper chamber, while PLC8024 (1 × 10^5^ cells) or Huh7 (1 × 10^5^ cells) were seeded in the bottom chamber. Forty-eight hours later, cells that invaded the bottom chamber were immobilized with a solution of paraformaldehyde, stained with 0.1% crystal violet, and counted under a microscope. For the inhibition assay, an MST1 inhibitor (Nanmu, Shanghai, China) (40 nM) was added to the bottom chamber and the assay was performed as described above.

### Enzyme-linked immunosorbent assay (ELISA) assay

The MST1 detection kit was purchased from JINGMEI Biotechnology (Jiangsu, China). ELISA was conducted according to the manufacturer’s instructions. The optical density (OD) values were determined using a spectrophotometer. The samples’ concentrations were ascertained by referencing the standard curve.

### Cell co-culture and flow assay

THP-1 cells, induced to adherent growth by phorbol 12-myristate 13-acetate (PMA, 100 ng/ml, ZhuanYan Biological Technology, Guangzhou, China), were co-cultured with conditioned medium (CM) from HCC cells for 48 h. The THP-1 cells were collected and subsequently washed with a PBS buffer three times. The cells were incubated with a primary antibody at room temperature for 15 min, followed by incubation with a secondary antibody and PBS washing. Flow cytometry analyses were carried out using Cyto-FLEX (Beckman Coulter, Fullerton, CA). The antibodies used are listed in the Supplementary Materials.

### Co-immunoprecipitation (Co-IP) assay

Co-IP assays were performed using a Pierce Direct Magnetic IP/Co-IP kit (Thermo Scientific, Rockford, IL) according to the manual. Briefly, cells were lysed and incubated on ice for 5 min with intermittent mixing. The lysate was quantified, and 500 µl of lysate was incubated with 25 µl of beads coupled with 5 µg of antibody at 4 °C overnight. Subsequent to thorough washing, proteins adherent to the beads were eluted. These proteins were then subjected to a western blotting procedure.

### Ubiquitination assay

Cells were treated with 10 μM MG-132 (Selleck Chemicals, Houston, TX) for 12 h to block proteasomal degradation. Cell lysates were collected and immunoprecipitated with anti-SKOR1 antibody (Immunoway, Plano, TX) (5 µg), eluted with 20 μL lysis buffer and denatured with SDS sample buffer (Thermo Scientific, Rockford, IL). Pull-down samples were subjected to immunoblotting with an anti-ubiquitin antibody (CST, Danvers, MA) to visualize polyubiquitylated SKOR1 protein bands.

### Cycloheximide chase assay

Cells were treated with 10 µM cycloheximide (MP Biomedicals, Santa Ana, CA), and total protein lysates were collected at different time points and subjected to immunoblotting analysis.

### Gelatin zymography assay

The activities of MMP2 and MMP9 were detected using an MMP Zymography assay kit (Applygen Technologie, Beijing, China). The cell protein extracts were mixed with an equal volume of 2×SDS-PAGE non-reducing buffer and electrophoresed on 8% SDS polyacrylamide gels containing 2 mg/ml of gelatin. Gels were then washed twice for 30 min in buffer A at room temperature, followed by incubation with buffer B for 4 h at room temperature. Gels were stained with 0.25% Coomassie brilliant blue and then destained in destaining buffer (10% acetic acid with 20% methanol). Finally, the images were taken under the Gel Doc XR+ (Bio-Rad, Hercules, CA).

### Statistical analyses

Statistical analyses were performed using SPSS software (version 23.0, Chicago, IL) and GraphPad Prism software (version 8.0.2, La Jolla, CA). A paired student’s *t*-test was performed to analyze the difference in mRNA levels between the two groups. Data are shown as mean ± SEM. *P* < 0.05 was considered statistically significant.

## Supplementary information

Supplementary materials

Original Date

## Data Availability

All data supporting the conclusions of the paper are available in the article and corresponding figures. The analysis of exon mutations of PDSS2 was performed using the publicly available TCGA Spliceseq. Clinical information for patients in TCGA_LIHC dataset were downloaded from cBioPortal database.
