# Supplementary material for: Overexpression of PDSS2-Del2 in HCC promotes tumor metastasis by interacting with macrophages
Source: Cell Death Discov. 2024 Dec 18;10:506. doi: 10.1038/s41420-024-02274-y (PMC11655556; doi:10.1038/s41420-024-02274-y)
Supplement: Supplementary file 1 — Supplementary materials [file 41420_2024_2274_MOESM1_ESM.docx]

**Supplementary information:**

**Primers :**

| Sequences of primers： | | |
| --- | --- | --- |
| PDSS2-Del2 | F: 5’ CTCTGCTTACCACAGCCAGT 3’  R: 5’ CACCTGCATAGCGATGTTGC 3’ |  |
| CXCL10 | F: 5’ GGTGAGAAGAGATGTCTGAATCC 3’  R: 5’ GTCCATCCTTGGAAGCACTGCA 3’ |  |
| CXCL13 | F: 5’ TATCCCTAGACGCTTCATTGATCG 3’  R: 5’ CCATTCAGCTTGAGGGTCCACA 3’ |  |
| IL-13 | F: 5’ ACGGTCATTGCTCTCACTTGCC 3’  R: 5’ CTGTCAGGTTGATGCTCCATACC 3’ |  |
| iNOS | F: 5’ GCTCTACACCTCCAATGTGACC 3’  R: 5’ CTGCCGAGATTTGAGCCTCATG 3’ |  |
| IL-1β | F: 5’ CCACAGACCTTCCAGGAGAATG 3’  R: 5’ GTGCAGTTCAGTGATCGTACAGG 3’ |  |
| 18S | F: 5’ GTAACCCGTTGAACCCCATT 3’  R: 5’ CCATCCAATCGGTAGTAGCG 3’ |  |
| CD163 | F: 5’ CCAGAAGGAACTTGTAGCCACAG 3’  R: 5’ CAGGCACCAAGCGTTTTGAGCT 3’ |  |
| CD206 | F: 5’ AGCCAACACCAGCTCCTCAAGA 3’  R: 5’ CAAAACGCTCGCGCATTGTCCA 3’ |  |
| ARG1 | F: 5’ TCATCTGGGTGGATGCTCACAC 3’  R: 5’ GAGAATCCTGGCACATCGGGAA 3’ |  |
| IL-10 | F: 5’ TCTCCGAGATGCCTTCAGCAGA 3’  R: 5’ TCAGACAAGGCTTGGCAACCCA 3’ |  |
| MMP-2 | F: 5’ AGCGAGTGGATGCCGCCTTTAA 3’  R: 5’ CATTCCAGGCATCTGCGATGAG 3’ |  |
| MMP-9 | F: 5’ GCCACTACTGTGCCTTTGAGTC 3’  R: 5’ CCCTCAGAGAATCGCCAGTACT 3’ |  |
| CCL18 | F: 5’ GTTGACTATTCTGAAACCAGCCC 3’  R: 5’ GTCGCTGATGTATTTCTGGACCC 3’ |  |
| CSF | F: 5’ TGAGACACCTCTCCAGTTGCTG 3’  R: 5’ GCAATCAGGCTTGGTCACCACA 3’ |  |
| MST1 | F: 5’ TGGTGCTACACGATGGACCCAA 3’  R: 5’ GCCACACTTCTCAAACTGCACC 3’ |  |
| SKOR1 | F: 5’ CGCCGATGATTTGGAAACGAGG 3’  R: 5’ CCTGTGACATCCAAGGTAAGCC 3’ |  |
| SMAD3 | F: 5’ TGAGGCTGTCTACCAGTTGACC 3’  R: 5’ GTGAGGACCTTGTCAAGCCACT 3’ |  |

**Antibodies:**

| **Antibody** | **Company** | **Catalog No.** |
| --- | --- | --- |
| PDSS2 | Wolwo |  |
| β-TUBULIN | CST | 86298S |
| MST1 | ABCLONAL | WH164977 |
| SKOR1 | IMMUNOWAY | yn3314 |
| GAPDH | PROTEINTECH | 10494-1-AP |
| SMAD3 | CST | 9513S |
| p-SMAD3 | CST | 9520S |
| ARG1 | BOSTER | A01106 |
| AKT | CST | 4691 |
| p-AKT | CST | 4060S |
| PI3K | CST | 4257S |
| C-RAF | CST | 9422S |
| MMP2 | CST | 40994S |
| MMP9 | CST | 13667S |
| CD163 | BioLegend | 333617 |
| CD206 | BioLegend | 141705 |
| CD68 | ABCAM | Ab955-500 |
