# Supplementary figures and images for: Overexpression of PDSS2-Del2 in HCC promotes tumor metastasis by interacting with macrophages
Source: Cell Death Discov. 2024 Dec 18;10:506. doi: 10.1038/s41420-024-02274-y (PMC11655556; doi:10.1038/s41420-024-02274-y)

Figure 2

C

C

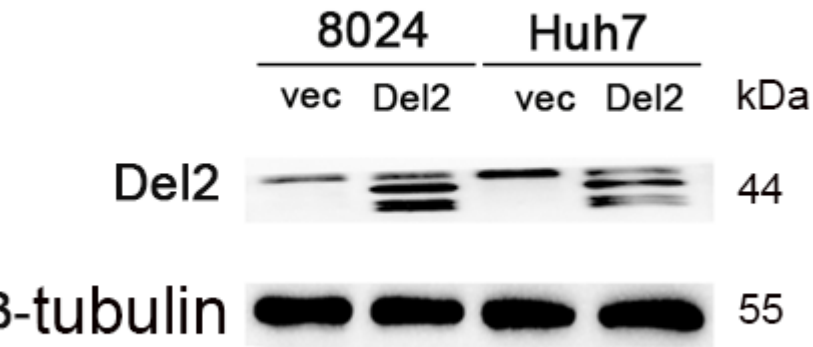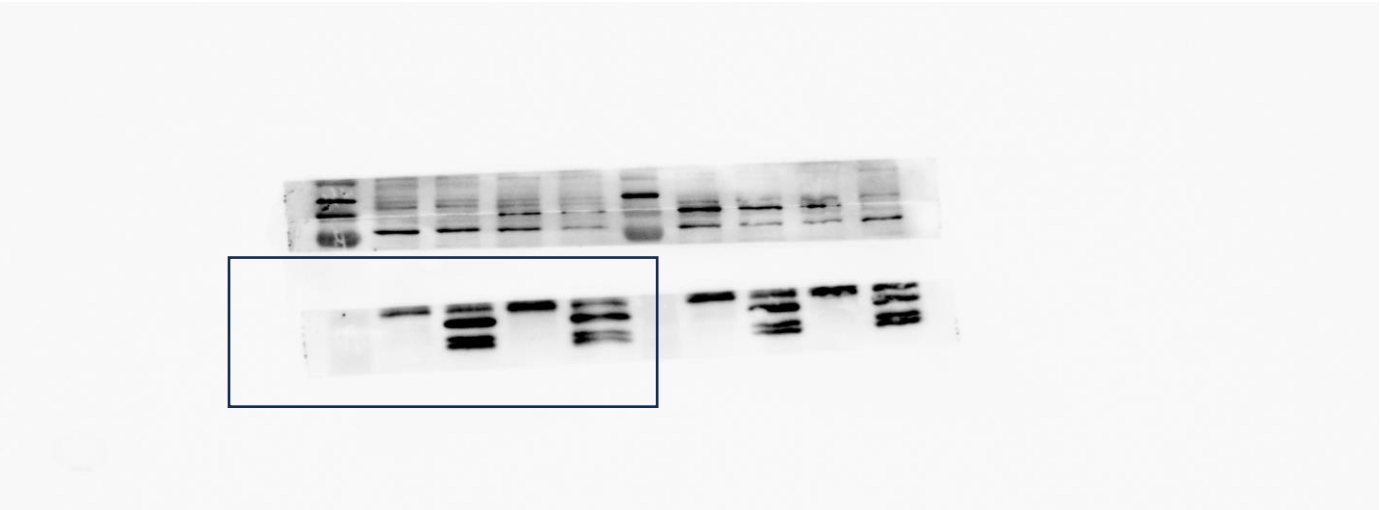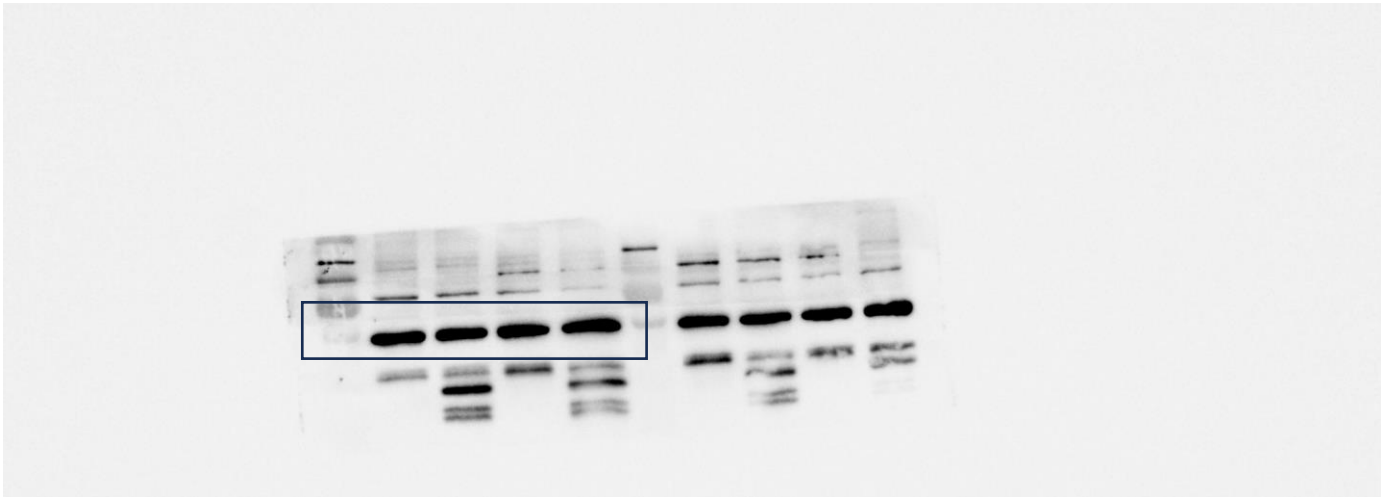

Figure 3

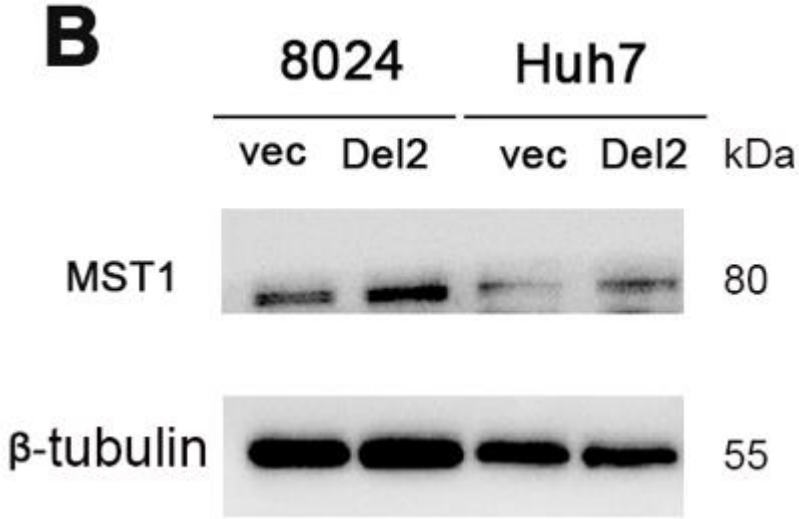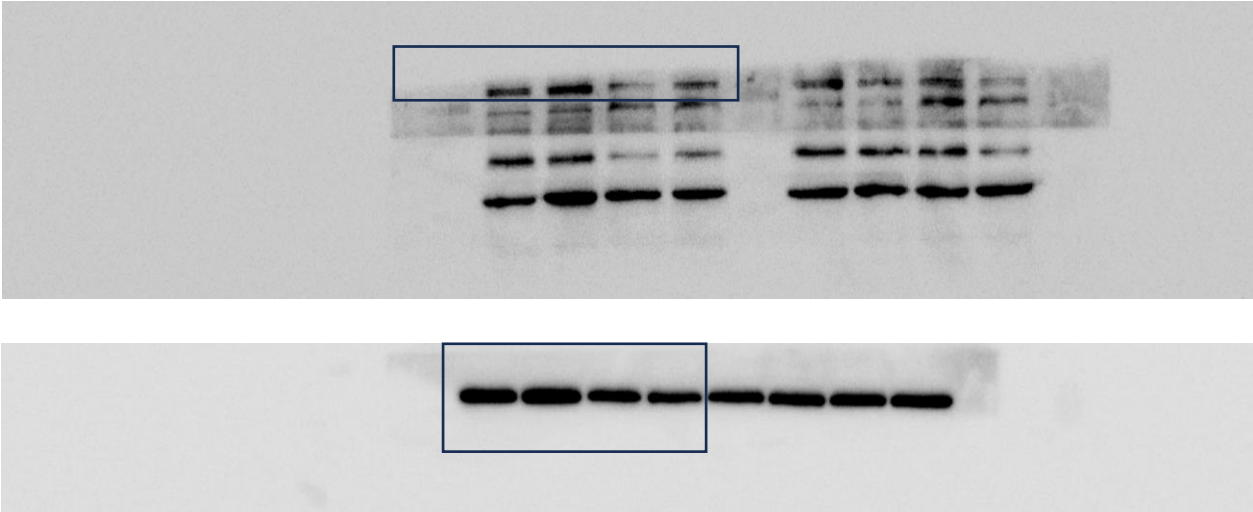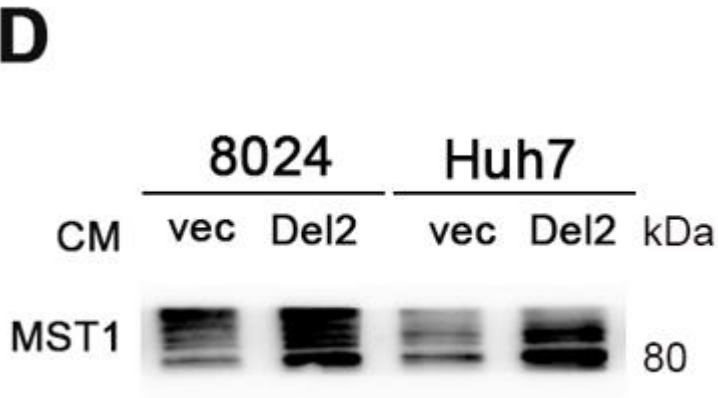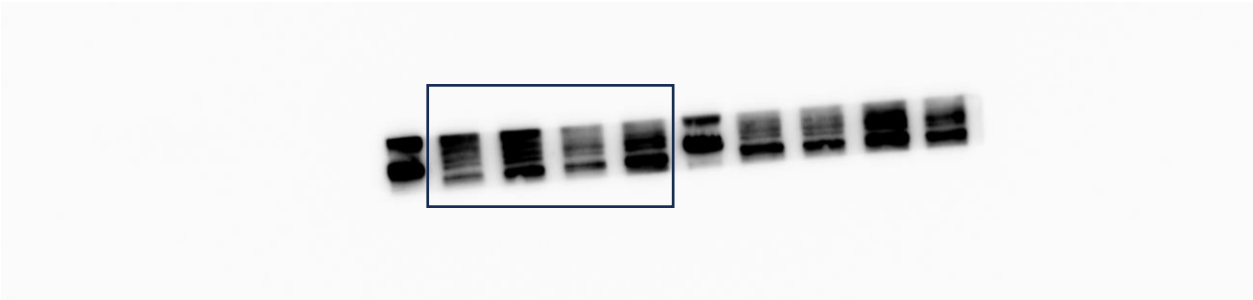

Figure 4

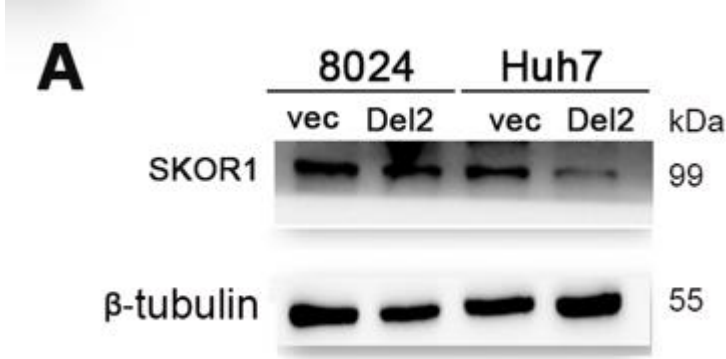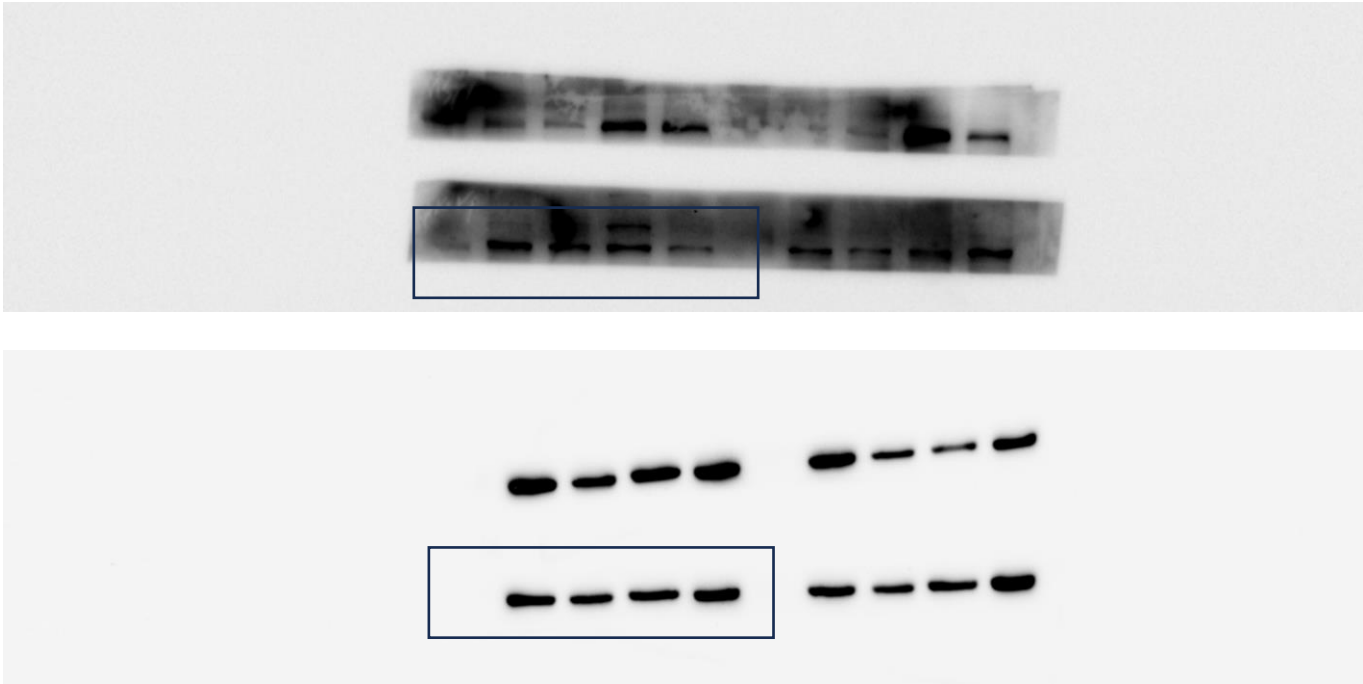

Figure 4

C

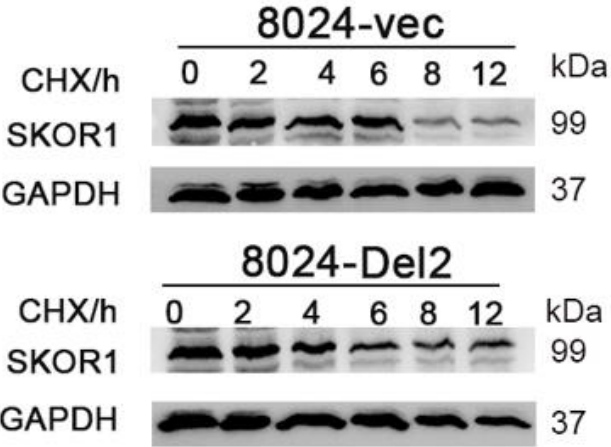

D

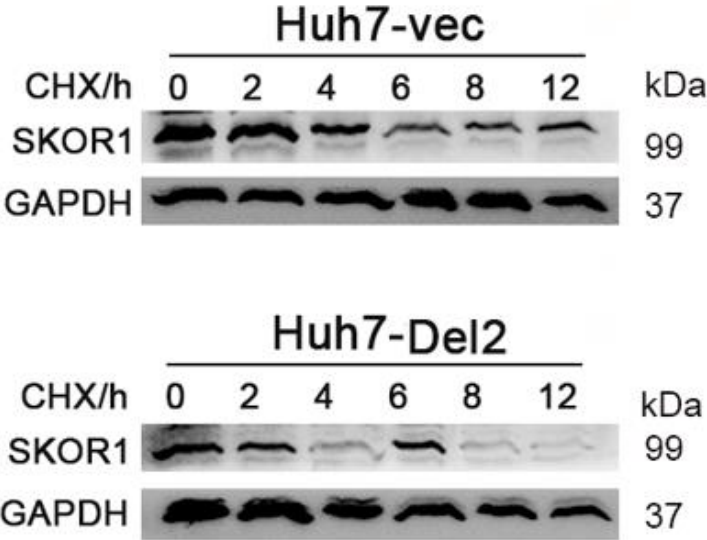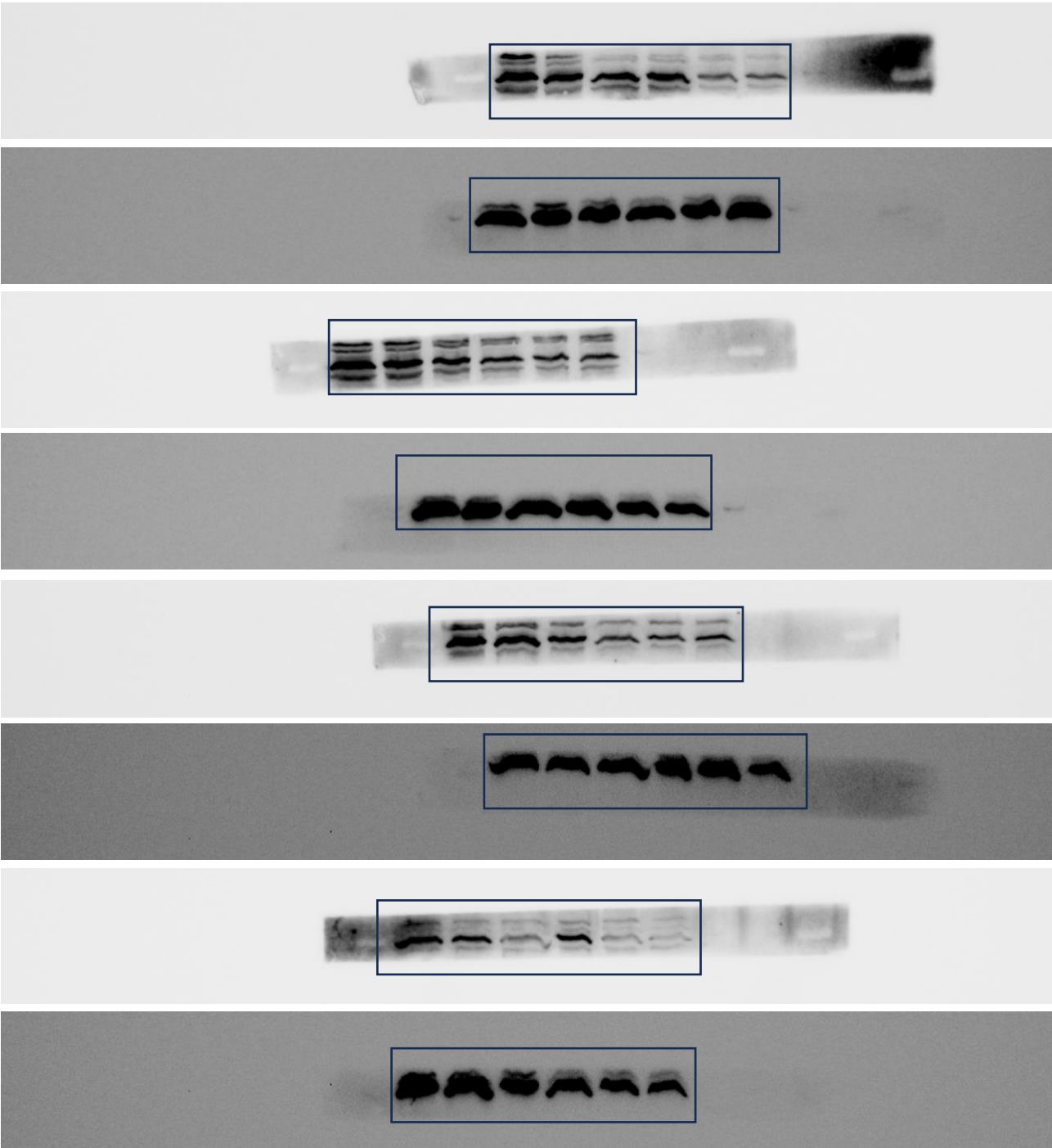

### Figure 4

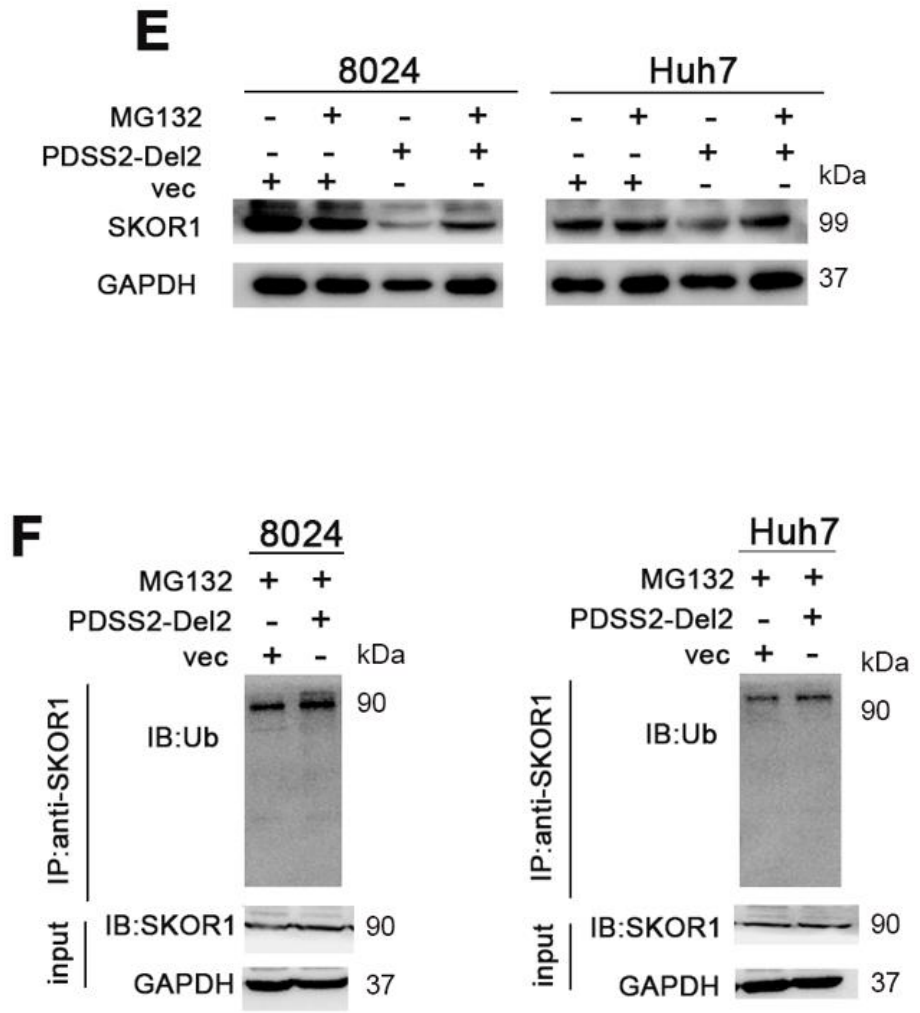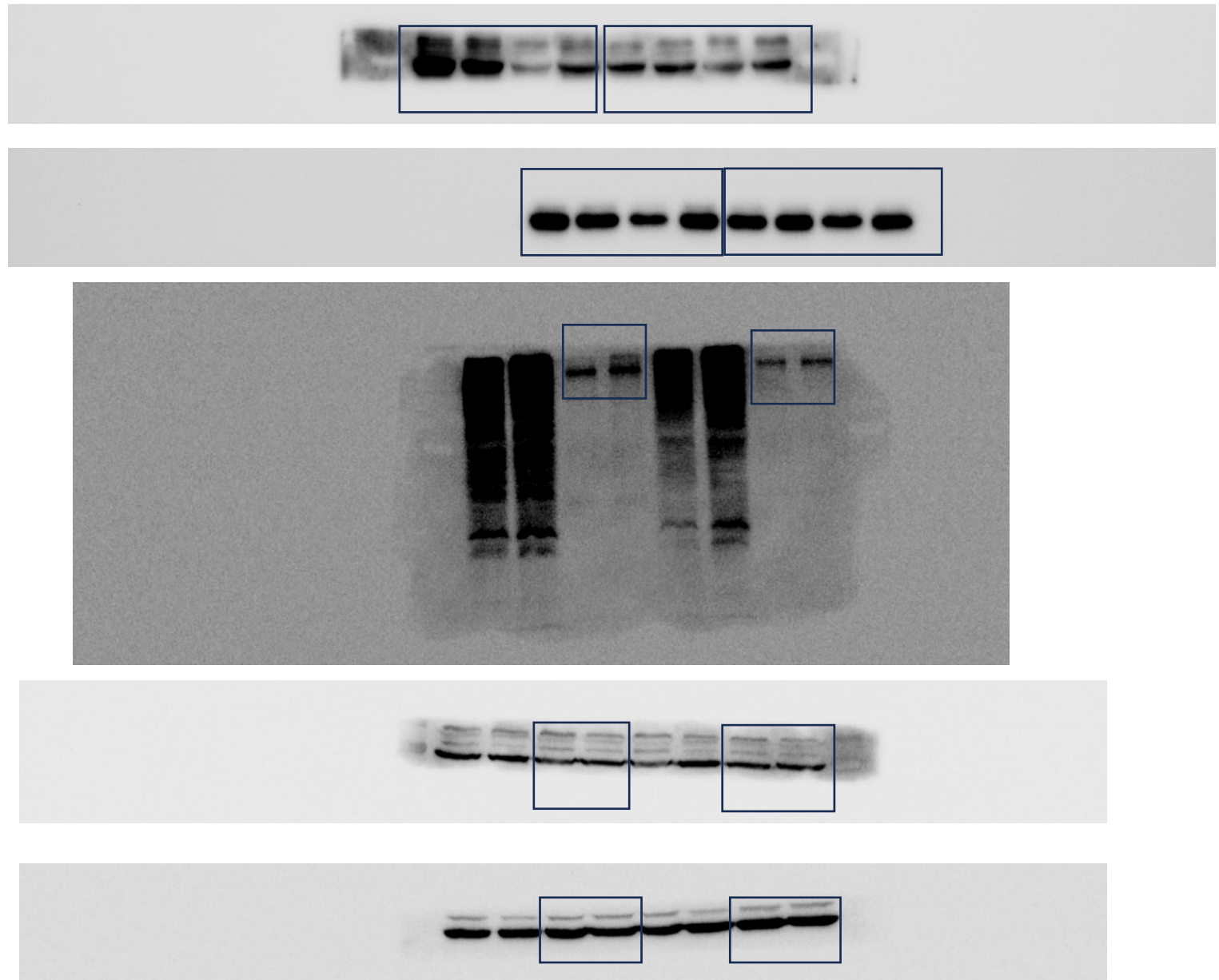

Figure 4

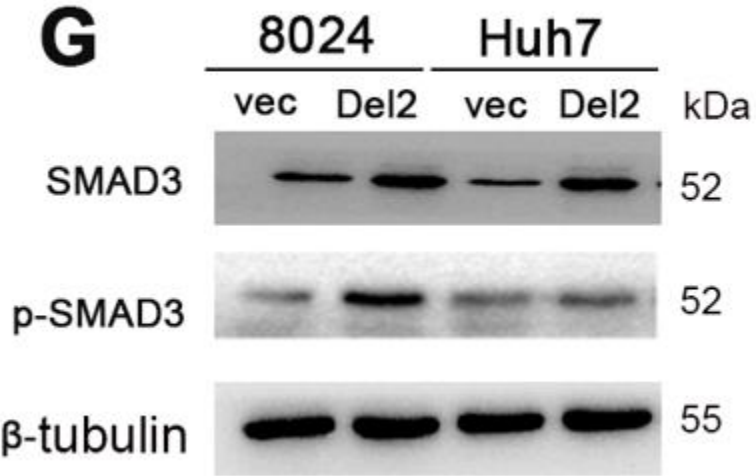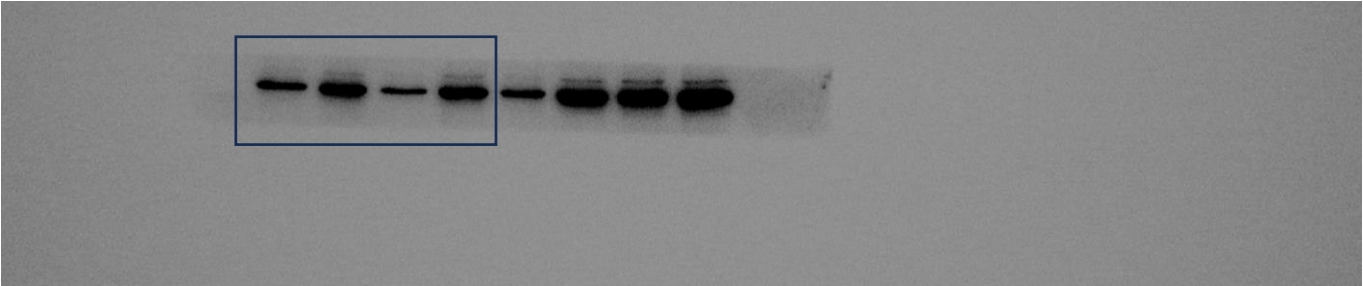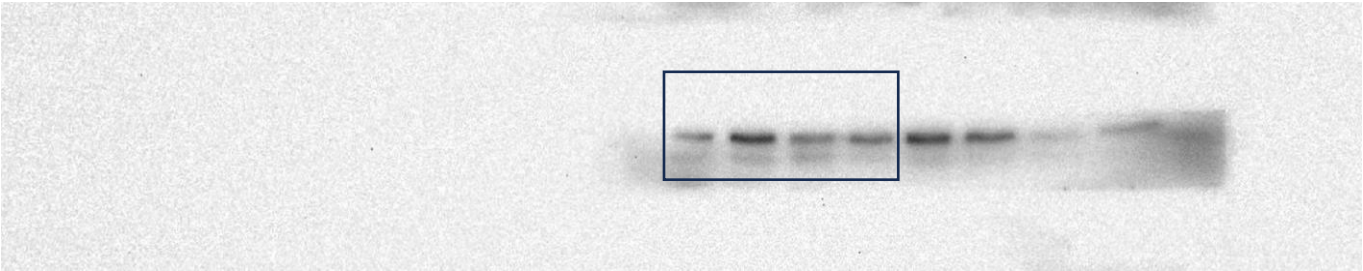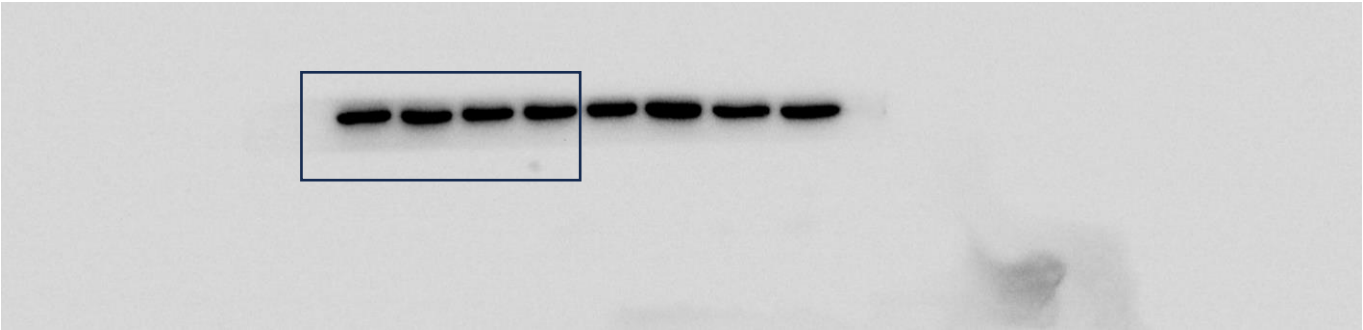

Figure 4

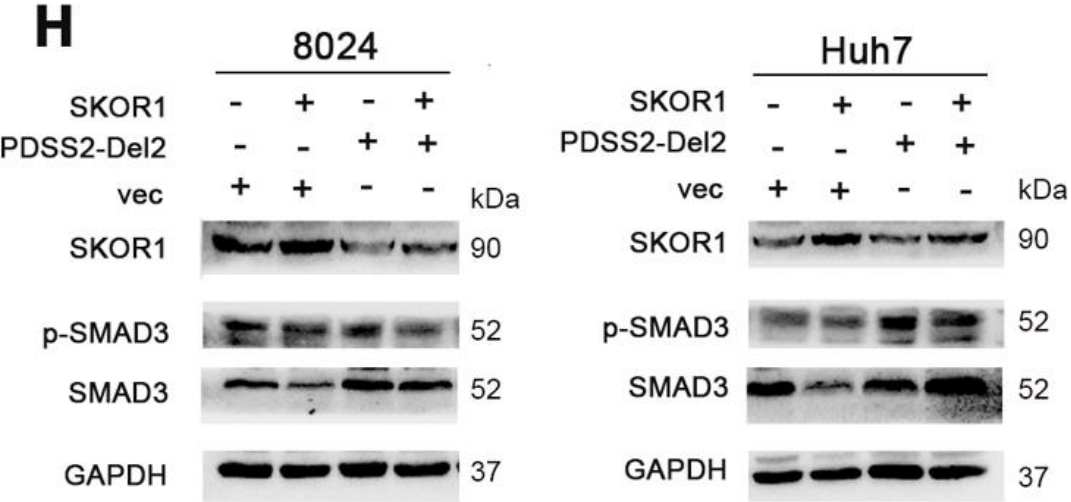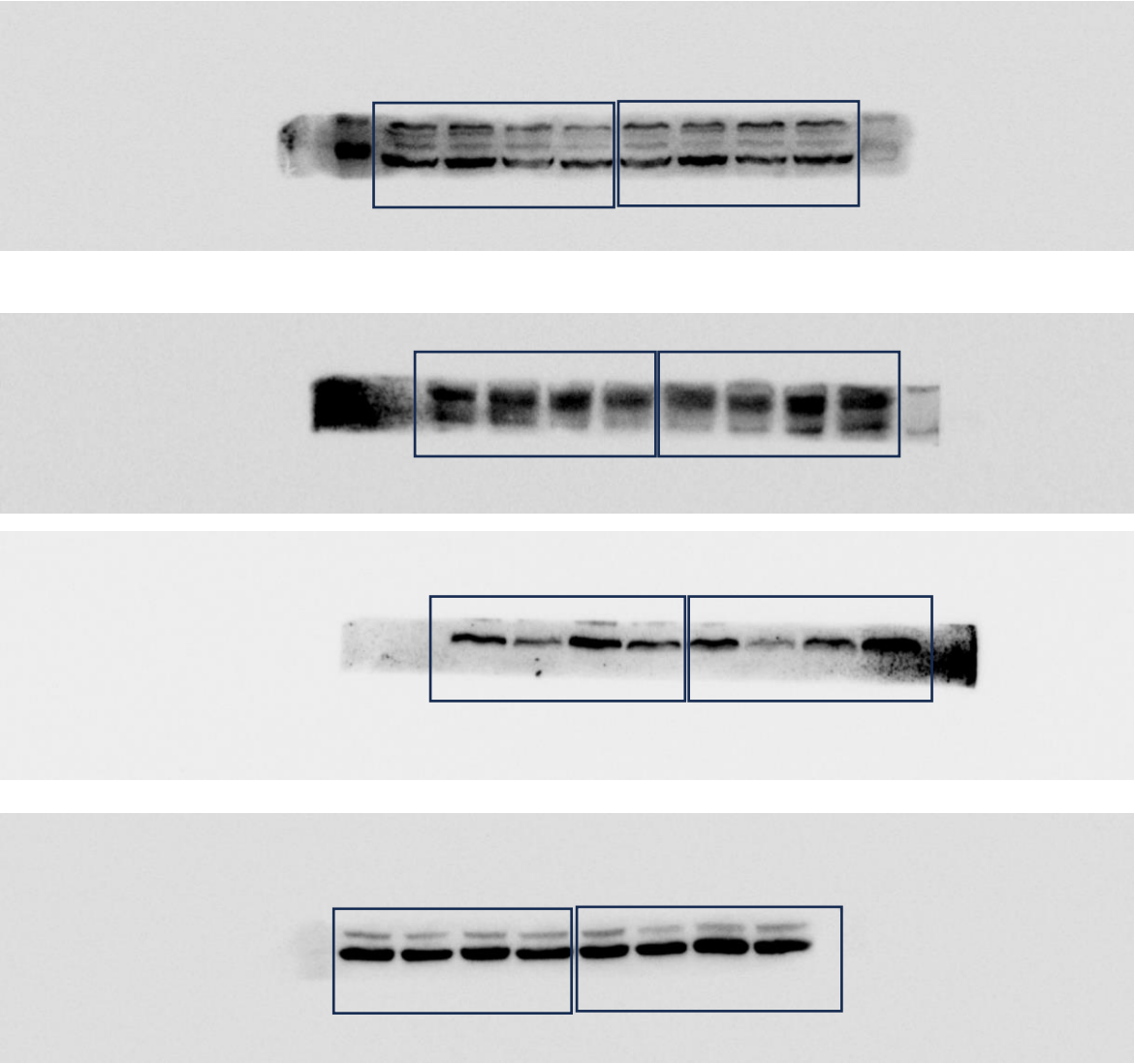

Figure 4

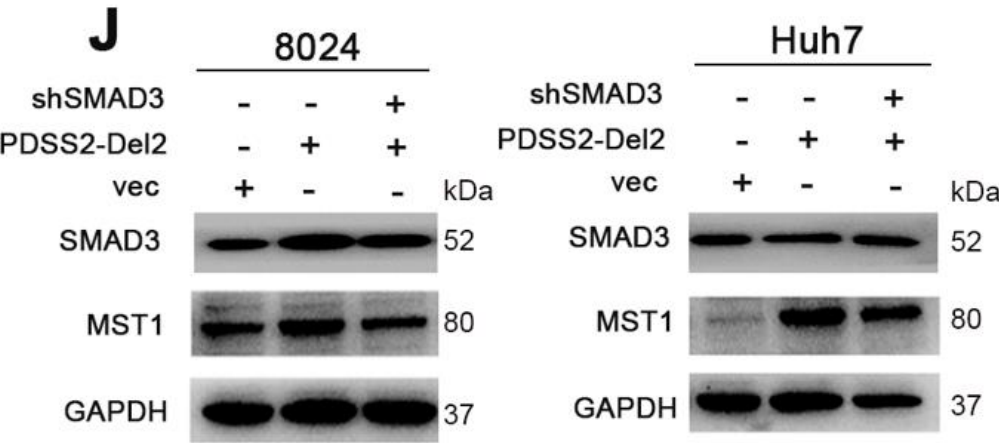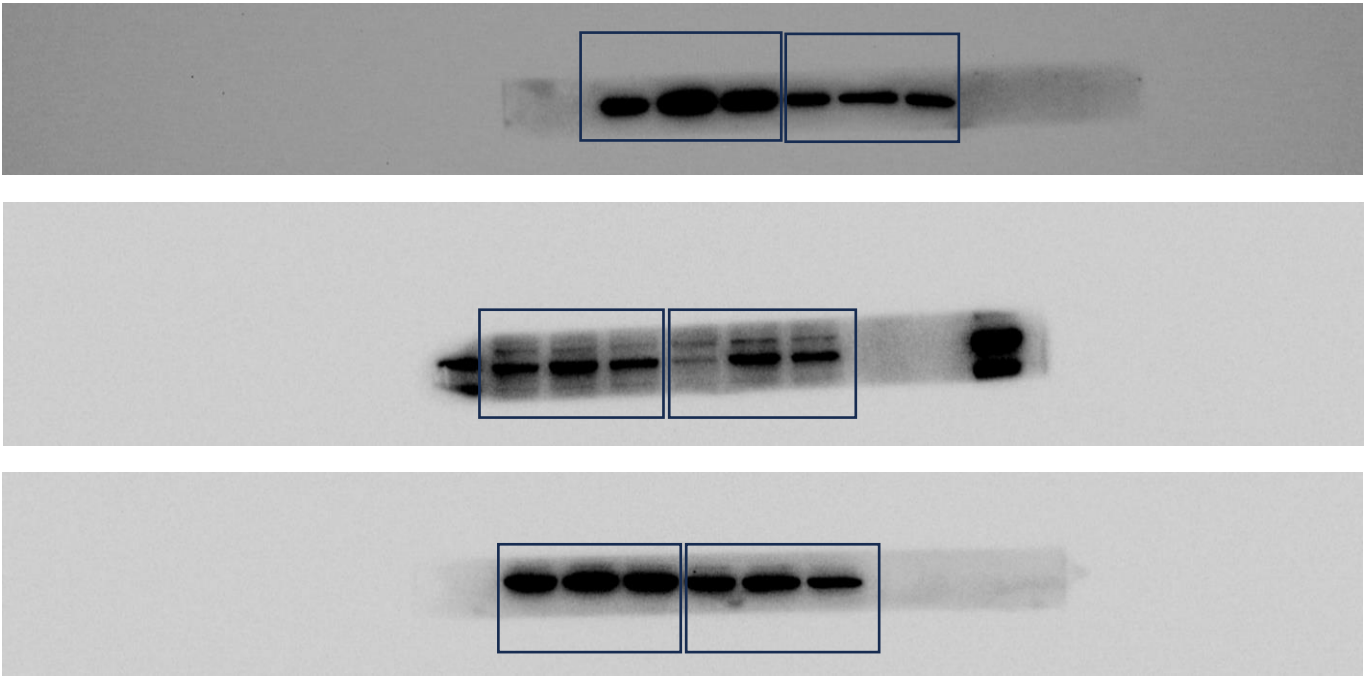

Figure 5

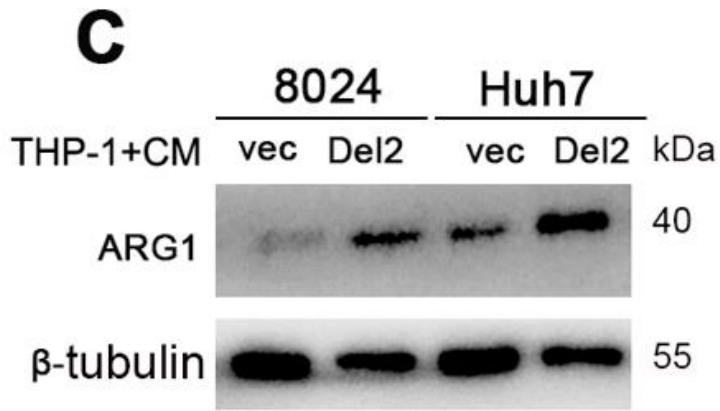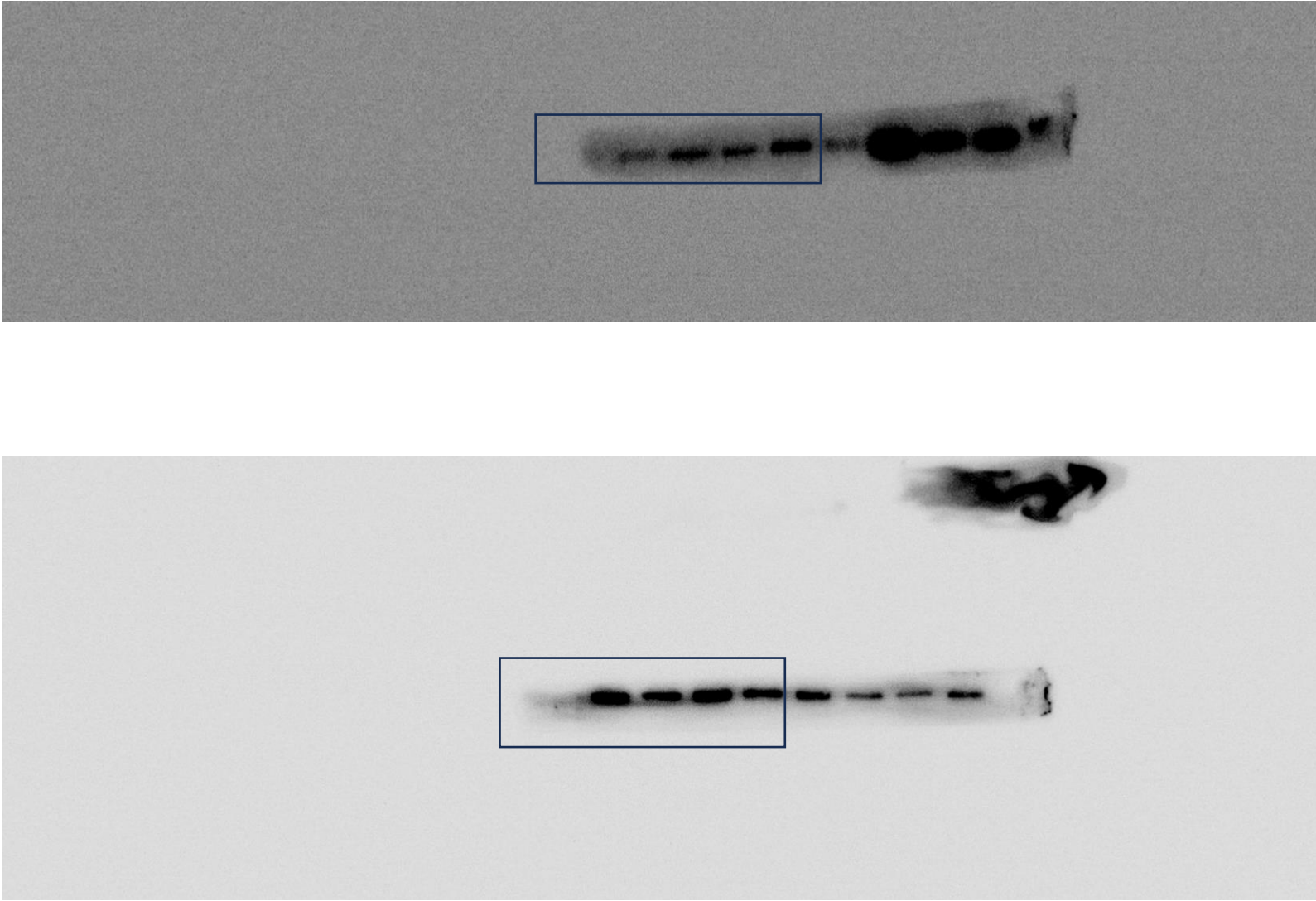

Figure 6

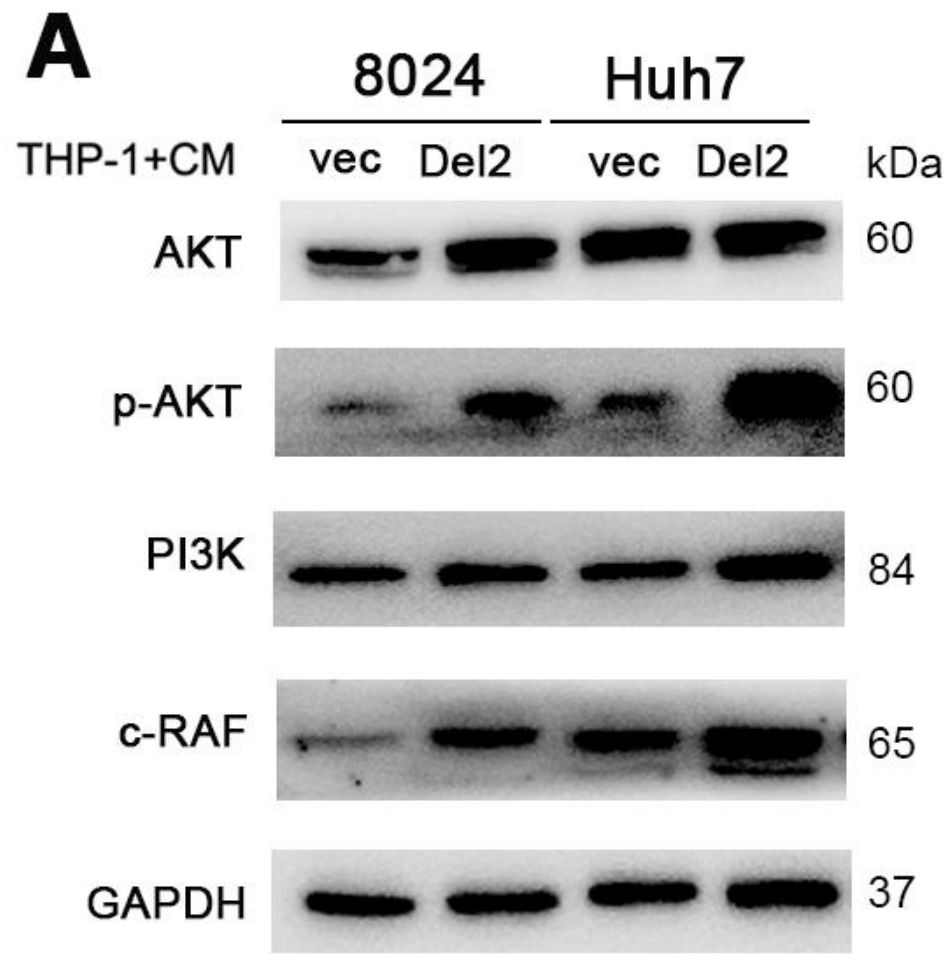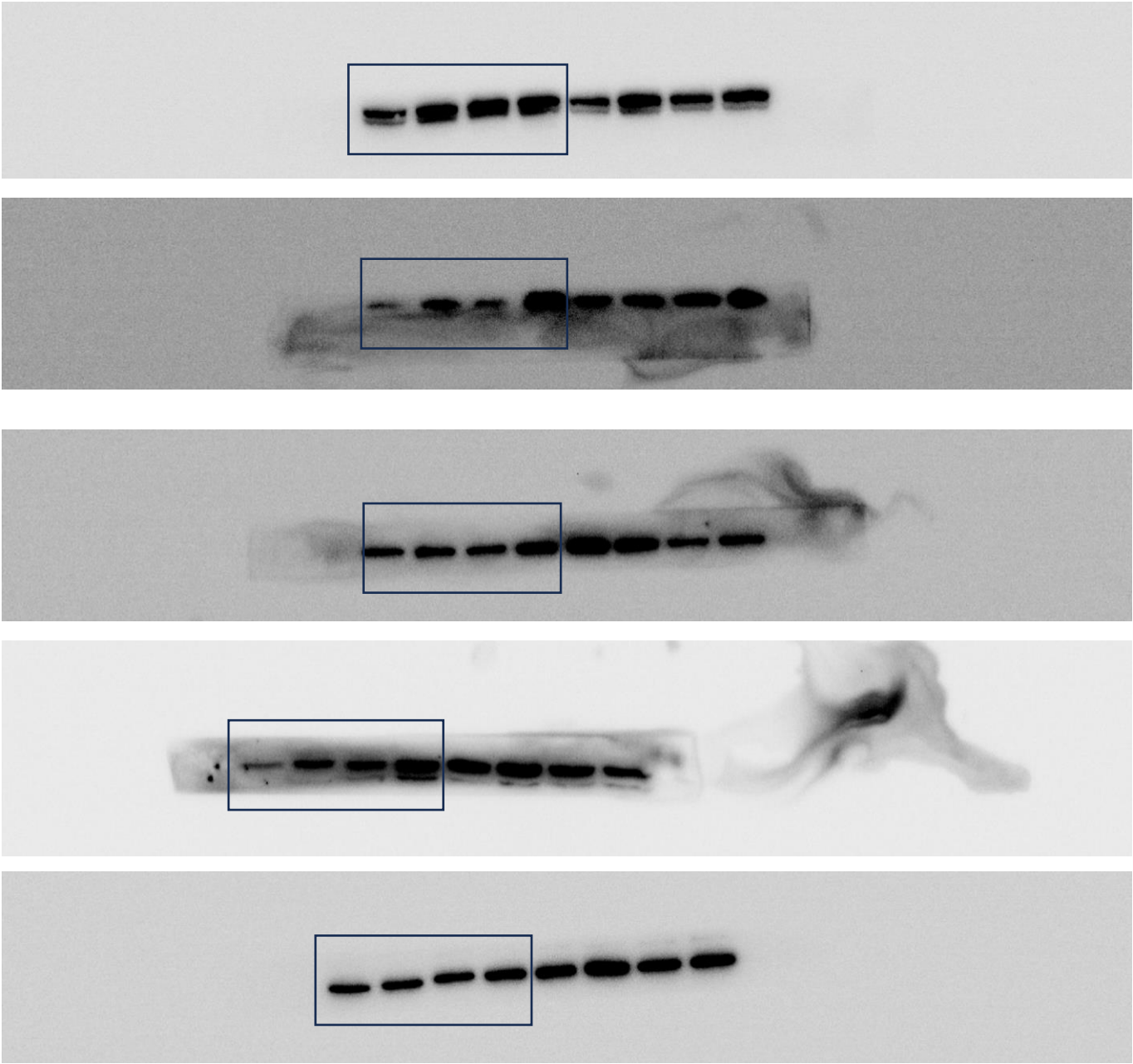

Figure 6

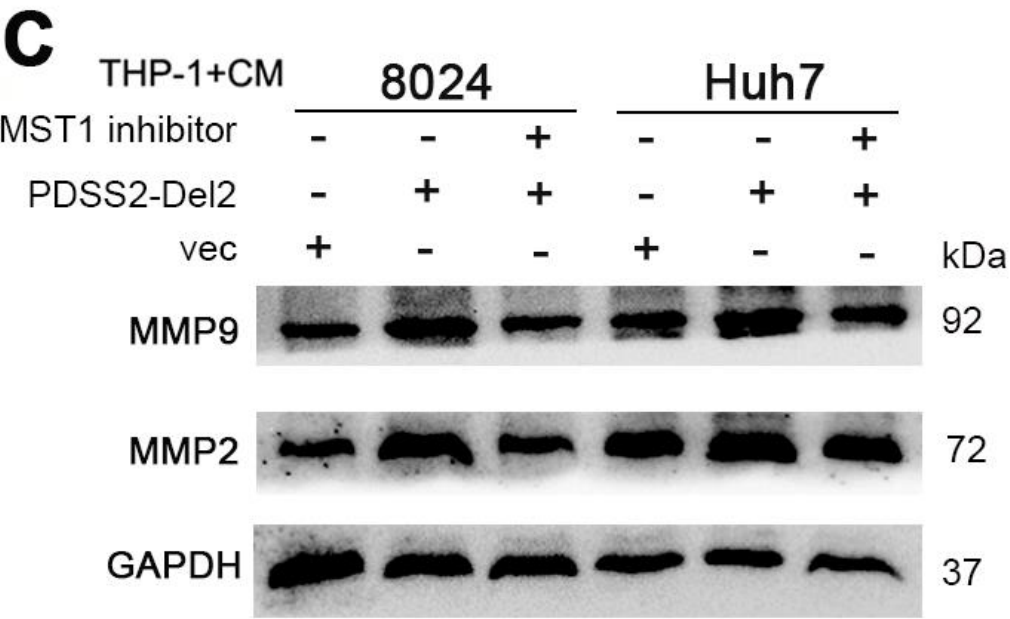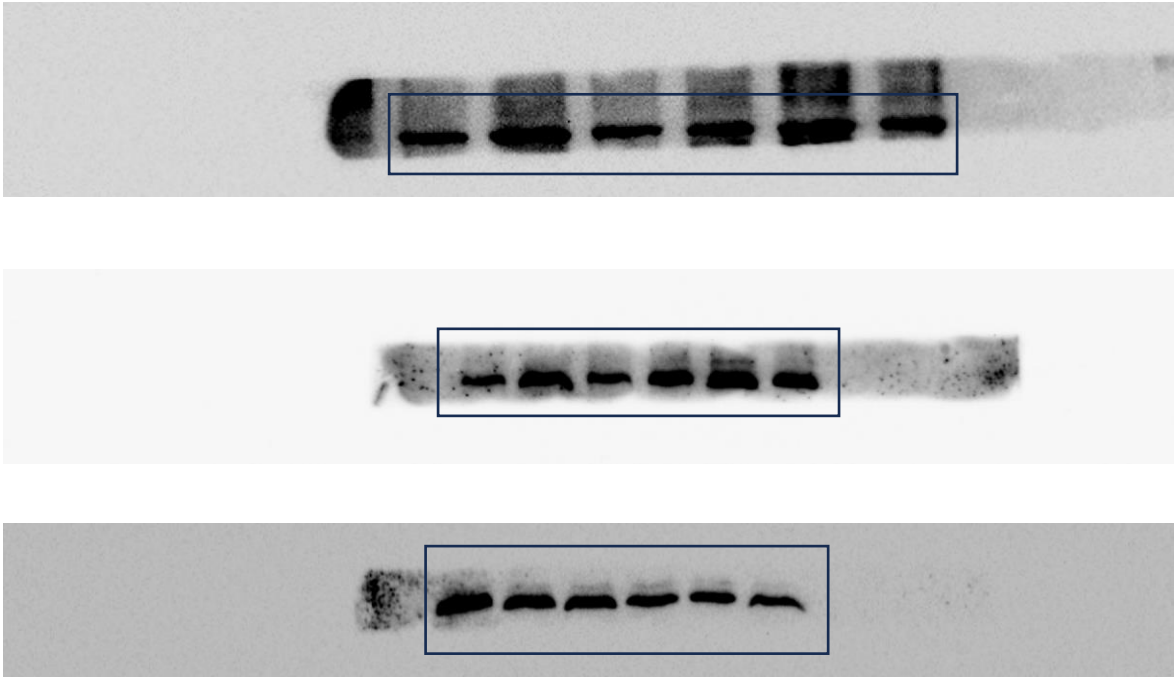

Figure 6

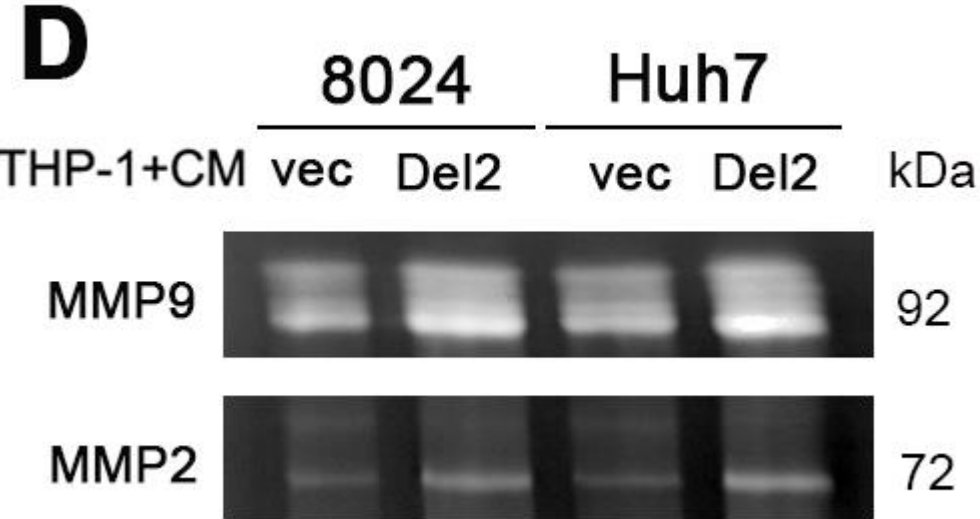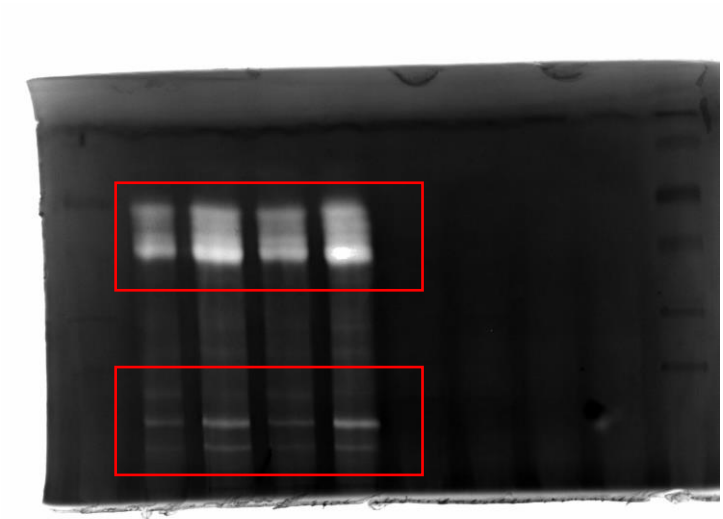

Supplement: Supplementary file 2 — Original Date [file 41420_2024_2274_MOESM2_ESM.pdf]
